# NOTCH1 signaling is dysregulated by loss of the deubiquitinase USP28 with del(11q), uncovering USP28 inhibition as novel therapeutic target in CLL

**DOI:** 10.1038/s41375-025-02632-4

**Published:** 2025-06-02

**Authors:** Alena Sophie Ehrmann, Miguel Quijada-Álamo, Viola Close, Min Guo, Valentina Carracoi, Claudia Pérez-Carretero, Luis Antonio Corchete, Tobias Friedrich, Benedetto Daniele Giaimo, Deyan Yordanov Yosifov, Johannes Bloehdorn, Alberto Rodríguez-Sánchez, Eugen Tausch, Christof Schneider, Hartmut Döhner, Thomas Kietzmann, Tilman Borggrefe, Stephan Stilgenbauer, Franz Oswald, Jesús-María Hernández-Rivas, Daniel Mertens

**Affiliations:** 1 University Hospital Ulm, Department of Internal Medicine III, Albert-Einstein-Allee 23, 89081 Ulm, Germany; 2 German Cancer Research Center (DKFZ), Bridging Group Mechanisms of Leukemogenesis, B061, Im Neuenheimer Feld 280, 69120 Heidelberg, Germany; 3 University of Salamanca, Department of Medicine, University Hospital of Salamanca, IBSAL, IBMCC, CSIC, Cancer Research Center, Campus Miguel de Unamuno s/n, 37007 Salamanca, Spain; 4 Icahn School of Medicine at Mount Sinai, Department of Oncological Sciences, New York, NY 10029 USA; 5 University Hospital Ulm, Department of Internal Medicine I, Albert-Einstein-Allee 23, 89081 Ulm, Germany; 6 Justus-Liebig-University Giessen, Institute of Biochemistry, Friedrichstrasse 24, 35392 Giessen, Germany; 7 University of Oulu, Faculty of Biochemistry and Molecular Medicine and Biocenter Oulu, Aapistie 7C, 90014 Oulu, Finland

**Keywords:** Chronic lymphocytic leukaemia, Targeted therapies, Cell signalling, Oncogenes, Chronic lymphocytic leukaemia

## Abstract

Aberrant active NOTCH1 signaling is a key pathogenic factor in chronic lymphocytic leukemia (CLL), detectable in half of patients and associated with disease progression. While some cases of active NOTCH1 signaling can be explained by mutations in *NOTCH1* or its regulators, like *FBXW7*, alternative mechanisms remain elusive. Here, we identified the deubiquitinase USP28 as regulator of NOTCH1 signaling in CLL. Notably, *USP28* is located within the frequently deleted chr11q23 region and is deleted in 90% of del(11q) patients, resulting in its decreased expression. USP28 interacts with the NOTCH1 intracellular domain (NICD) independently of FBXW7 and the NICD-PEST domain, stabilizing NICD and enhancing NOTCH1 signaling. Integrating RBPJ-occupied genes in HG3 cells, RNA-Seq of *USP28*^*WT*/*KO*^ cells and gene expression from del(11q) CLL patients, we identified 15 NOTCH1 target genes specifically dysregulated by deletion of USP28 and del(11q) potentially influencing CLL pathogenesis. Pharmacological inhibition of USP28 with the small molecule AZ1 suppressed NOTCH1 activation in primary CLL cells. AZ1 combined with the BCL-2 inhibitor venetoclax reduced CLL cell viability, particularly in samples with high NOTCH1 activity. Our findings highlight USP28 as promising therapeutic target and provide a rationale for combined inhibition of USP28 and BCL-2 in CLL patients with active NOTCH1 signaling.

## Introduction

Chronic lymphocytic leukemia (CLL) is a prevalent B-cell malignancy characterized by diverse clinical courses of disease progression and treatment response, influenced by various genetic abnormalities. Besides chromosomal aberrations, such as deletion of 17p, deletion of 13q, deletion of 11q (del(11q)) and trisomy 12, gene mutations in *TP53*, *ATM*, *SF3B1*, *NOTCH1* and others are common [1–4]. These mutations lead to the dysregulation of biological pathways that are involved in the development and survival of B-cells [2].

To date, CLL is incurable but well treatable with drugs inhibiting different components of the B-cell receptor (BCR) signaling pathway or the anti-apoptotic protein BCL-2. However, refractory disease and treatment resistance occur mainly through transformation of the disease in secondary lymphoid organs, suggesting that the microenvironment within the lymph nodes is crucial for CLL cell survival. One key microenvironmental integrator is the NOTCH1 signaling pathway. Mutations in *NOTCH1* itself or in NOTCH1 regulators and active NOTCH1 signaling in CLL are predictive factors for dismal prognosis and poor response to anti-CD20 antibodies used in the therapy of CLL [5–9].

The prevalence of *NOTCH1* mutations is approximately 6–12% in CLL patients at diagnosis and higher in patients with progressive disease [10, 11]. Most CLL-related *NOTCH1* mutations are located in exon 34 and the 3’ untranslated region (UTR), resulting in truncation of NOTCH1 by deletion of the C-terminal PEST (proline, glutamine, serine and threonine-rich) domain, which regulates degradation of active NOTCH1 [12, 13]. Upon activation of the NOTCH1 pathway, the NOTCH1 intracellular domain (NICD) is cleaved from the NOTCH1 receptor at the cell membrane. The NICD translocates to the nucleus where, together with co-factors, it initiates transcription of target genes [14, 15]. Following this, the NOTCH1 signaling cascade is deactivated by degradation of NICD, initiated by phosphorylation of the so called CDC4-phosphodegron (CPD) motif within the PEST domain, which in turn leads to ubiquitination mediated by the E3-ubiquitin ligase FBXW7 and proteasomal degradation [16]. In *NOTCH1* mutated CLL cells, deletion of the PEST domain impairs NICD degradation resulting in increased NOTCH1 signaling activity [3, 13]. Interestingly, 50% of CLL patients with active NOTCH1 signaling do not have *NOTCH1* mutations [14]. A subset of these patients exhibits genetic alterations in modulators of the NOTCH1 pathway such as *FBXW7*, *MED12* or *SPEN* [17–20]. However, these mutations cannot explain all CLL cases with activated NOTCH1 signaling, suggesting additional mechanisms of NOTCH1 activation in CLL [14]. CLL patients with mutations in regulators of NOTCH1 signaling show similar clinical outcome to patients with *NOTCH1* mutations [7]. However, pharmacological inhibition of NOTCH1 itself has not yet been proven to be a successful therapeutic option [21]. It is therefore important to identify the mechanisms responsible for NOTCH1 activation in CLL to discover new therapeutic options for patients with hyperactive NOTCH1 signaling even without *NOTCH1* mutations.

Importantly, additional mechanisms activating NOTCH1 signaling might involve dysregulation of NICD degradation via the ubiquitin-proteasome system. Specifically, the FBXW7-mediated ubiquitination of NICD can be counteracted by the ubiquitin specific protease 28 (USP28) [22–24]. USP28 is a deubiquitinase that is found upregulated in solid tumors like colorectal cancer, squamous cell carcinoma and breast cancer. In these cancers, USP28-mediated deubiquitination stabilizes important oncogenes including c-MYC, ∆Np63, c-JUN, HIF-1α and NICD indicating an oncogenic function for USP28 [25–29]. Interestingly, *USP28* is located on chromosomal band 11q23, in proximity to the *ATM* gene locus. This region is frequently deleted in CLL [1, 30, 31]. However, the biological role of USP28 in CLL, including its relation to NOTCH1 signaling, has not been investigated so far.

In this study, we explored the role of USP28 in the regulation of NOTCH1 signaling in CLL. By analyzing the effect of USP28 deletion or overexpression in primary CLL cells and CRISPR/Cas9-generated del(11q) or *USP28*^*WT/KO*^ CLL cell lines, we found that NOTCH1 activity is regulated via interaction with USP28. Furthermore, pharmacological inhibition of USP28 with the small molecule AZ1 downregulated NOTCH1 activity and NICD protein levels, leading to decreased viability of primary CLL cells. These findings highlight USP28 as a potential therapeutic target for CLL patients with active NOTCH1 signaling.

## Methods

Detailed method descriptions of cell line generation and culture conditions, treatment compounds, luciferase assays, western blotting, immunofluorescence microscopy, immunoprecipitations, quantitative real time PCR and viability experiments are available in the supplemental material.

### Primary CLL cells

Primary cells from CLL patients were collected at the University Hospitals of Salamanca (Spain) and Ulm (Germany) after written informed consent (Ulm Ethics Committee, Vote 242/20). PBMCs were obtained from heparinized peripheral blood either by Ficoll gradient centrifugation or by using the human B-CLL cell isolation kit (Miltenyi Biotec, Bergisch Gladbach, Germany). Cells were viably preserved in liquid nitrogen until use.

### HG3 CRISPR/Cas9 cell lines

Cas9-expressing HG3 cells (HG3-Cas9), HG3-del(11q) and HG3-del(11q) *ATM*^*KO*^ cell lines were previously generated and tested for Cas9 activity [32]. Generation of HG3 CRISPR/Cas9 cell lines with monoallelic *USP28* deletion (*USP28*^*WT/KO*^) is described in more detail in the supplemental material.

### RNA-Seq and ChIP-Seq

HG3 *USP28*^*WT/WT*^ (*n* = 2) and HG3 *USP28*^*WT/KO*^ (*n* = 2) clones were analyzed by RNA-Seq following the TruSeq Stranded mRNA protocol (Illumina, San Diego, CA, USA). ChIP-Seq and CUT&Tag of HG3 cells was performed as described previously [33]. More details are available in the supplemental material.

## Results

### USP28 is deleted in del(11q) patients resulting in decreased NOTCH1 signaling

**Fig. 1:**
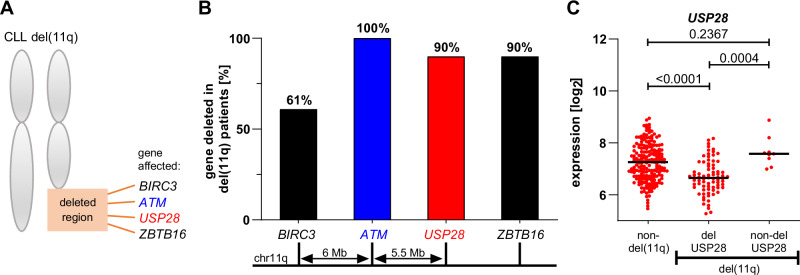
USP28 is deleted and downregulated by del(11q) in CLL. **A** Schematic representation of genes that are affected by deletion in del(11q) CLL cases. Included genes in the region: *BIRC3, ATM, USP28* and *ZBTB16*. **B** Proportion of monoallelic gene deletion of *BIRC3, ATM, USP28* and *ZBTB16* among 96 del(11q) cases from high-resolution SNP-array data [34]. **C** *USP28* expression in a clinical trial cohort of CLL patients (CLL8). Comparison between patients with (*n* = 86) and without (*n* = 199) del(11q). The gene expression data was generated from CD19^+^ sorted CLL cell samples [35]. Lines represent the median. Statistical significance was determined by Kruskal–Wallis test followed by Dunn’s multiple comparison test.

The heterozygous deletion of 11q in CLL can affect several genes located in proximity to *ATM* including *USP28* (Fig. 1A). To identify candidate genes in this region that possibly contribute to the pathomechanism of CLL, we analyzed data from SNP array and gene expression profiling from CLL patients of the CLL8 study cohort [34, 35]. *ATM* and *BIRC3* have already been reported to play a role in del(11q) CLL and were deleted in 100% and 61% of del(11q) patients, respectively. Interestingly, we identified *USP28* and *ZBTB16* being deleted in 90% of del(11q) patients and thus deleted at an even higher frequency than *BIRC3* (Fig. 1B). The deletions of *ATM, BIRC3* and *USP28* but not *ZBTB16* resulted in significantly decreased mRNA expression levels (Fig. 1C and Supplementary Fig. 1A). This highly recurrent deletion of *USP28* that coincides with a downregulation of *USP28* mRNA levels substantiates a possible role of USP28 loss in the dysregulation of signaling pathways that contribute to the pathobiology and disease course of del(11q) CLL.

**Fig. 2:**
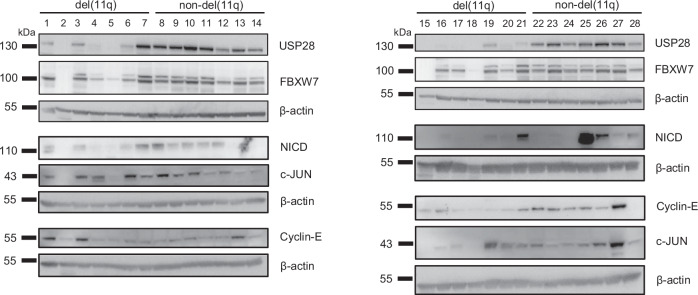
USP28 and its target proteins are downregulated in del(11q) CLL patients. Protein expression of USP28 and FBXW7 and their target proteins NICD, Cyclin-E and c-JUN in primary CLL cells with (*n* = 14) and without del(11q) (*n* = 14). Additional 8 patients and quantification of protein levels are available in Supplementary Fig. 1. Patient characteristics are displayed in Supplementary Table 1.

To further investigate the impact of del(11q) on USP28 and its function to prevent degradation of proteins, we analyzed protein levels of the previously identified FBXW7 target proteins NICD, Cyclin-E and c-JUN [23] in primary cells of CLL patients (Supplementary Table 1) harboring del(11q) (*n* = 18) and patients without del(11q) (non-del(11q), *n* = 18). USP28 protein levels were significantly reduced in del(11q) patient cells, correlating with significantly reduced NICD levels (Fig. 2 and Supplementary Fig. 1B and C). In addition, Cyclin-E and c-JUN exhibited slight, non-significant trends of downregulation in del(11q) patient cells compared to non-del(11q) patient cells (Fig. 2 and Supplementary Fig. 1B and C), suggesting that lower USP28 levels in del(11q) increase degradation of FBXW7 target proteins.

### USP28 interacts with NICD and affects NOTCH1 stability and activity

Next, we aimed to identify the mechanisms by which USP28 affects NOTCH1 signaling. USP28 can counteract FBXW7-mediated degradation of NICD through deubiquitination, mediated by its interaction with the CPD motif within the NICD PEST domain. The CPD motif, when phosphorylated, marks NICD for either ubiquitination or deubiquitination [22–24]. Moreover, phosphorylation of USP28 itself at the specific serine (S) residues 67 and 714 has been shown to be an important aspect regulating the interaction of USP28 with target proteins and enhancing its deubiquitination activity [36, 37]. About the interaction of USP28 with its target c-MYC it was reported, that USP28 and c-MYC only interact in the presence of FBXW7 through a joint interaction [22]. In contrast, more recently it was shown that USP28 can interact with c-MYC in a FBXW7 independent manner, but only with unphosphorylated CPD motifs [26].

**Fig. 3:**
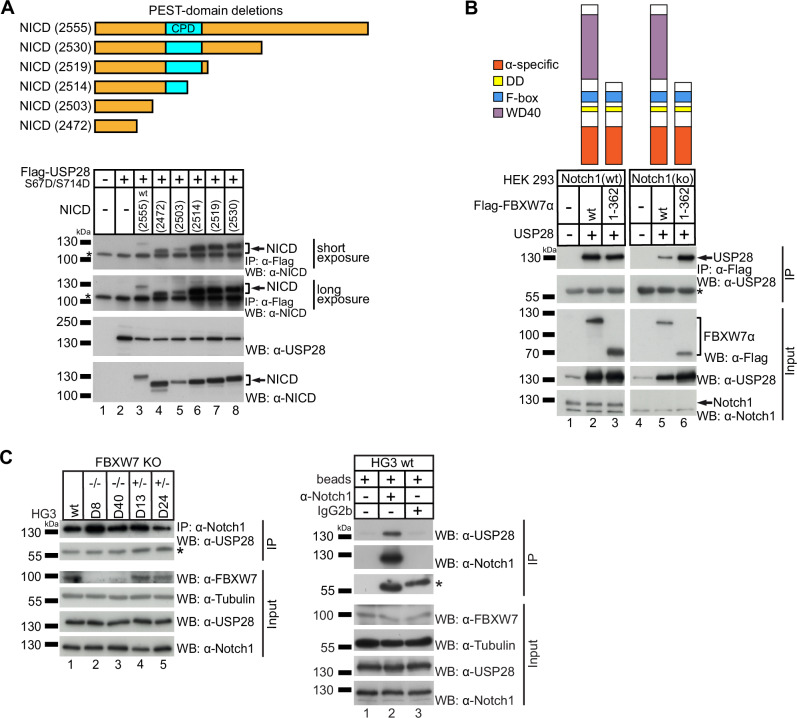
USP28 interacts with NICD independently of the NICD PEST domain and FBXW7. **A** HEK293 cells were co-transfected with FLAG-USP28 S67D/S714D and different NICD variants with different levels of truncation from the side of the C-terminus (upper panel). Co-immunoprecipitation (lower panel) was performed 24 h after transfection and analyzed by western blot of which a short (upper panel) and long (second panel from top) exposure are shown. Expression of the protein derived from the transfected constructs was detected via western blot shown in the two lower panels. * marks the heavy chain of the anti-FLAG antibody used for immunoprecipitation. **B** NOTCH1 wt (left panel) or NOTCH1 ko (right panel) HEK293 cells were transfected with the indicated FLAG-tagged FBXW7α constructs with complete or truncated WD40 domain which is important for interaction with NICD. Additionally, untagged USP28 was transfected. Co-immunoprecipitation was performed 24 h after transfection and analyzed via western blot shown in the upper panel (IP). The expression of the protein derived from the transfected constructs is shown in the lower panels (Input). * marks the heavy fragment of the anti-FLAG antibody used for IP. **C** Immunoprecipitation of endogenous NOTCH1 from HG3 wt or the HG3 CRISPR/Cas9-modified FBXW7 WD40 domain knockout cell lines D8, D40, D13 and D24 [17]. Co-immunoprecipitation of USP28 was analyzed via western blot (upper panel, IP). The expression of FBXW7, USP28 and NOTCH1 in the cell lines is shown in the lower panels (Input). Specificity of the endogenous NOTCH1 precipitation was confirmed by IP reactions with only beads or an IgG2b isotype control antibody. * marks the heavy chain of antibodies used for IP. Western blots are representative for at least three independently performed experiments. IP immunoprecipitation, WB western blot, wt wild type.

Thus, we aimed to clarify the mechanism of the USP28/NICD interaction, and determine the role of FBXW7 in this interaction. Immunofluorescence microscopy revealed that transfected FBXW7α, wild type (wt) USP28, a USP28 variant mimicking phosphorylation via aspartic acid (D) at S67 and S714 (USP28 S67D/S714D) and NICD are predominantly co-localized in the nucleus (Supplementary Fig. 2), suggesting a potential functional interaction. Co-immunoprecipitation (Co-IP) experiments confirmed the interaction of NICD with USP28 wt and the phosphomimetic USP28 S67D/S714D variant. Notably, the USP28 S67D/S714D variant showed stronger interaction with NICD compared to USP28 wt (Supplementary Fig. 3A). To prove whether the CPD motif is necessary for the interaction of USP28 and NICD, we performed further Co-IP experiments. As a result, we observed that the USP28 S67D/S714D variant was able to interact with NICD variants with different levels of truncation of the PEST domain, up to its almost complete deletion with full deletion of the CPD motif (Fig. 3A). This finding contradicts the hypothesis that USP28 relies on the CPD motif for the interaction with NICD.

In line with previous findings, we found that FBXW7 requires its WD40 domain for the interaction with NICD, as the interaction was lost with truncation of the WD40 domain or a specific hotspot mutation within the WD40 domain (R505C; Supplementary Fig. 3B) [17]. However, interaction of FBXW7 with USP28 was not impaired by deletion of the WD40 domain and was also shown in the absence of NOTCH1 in HEK293 *NOTCH1* knockout cells (Fig. 3B). Finally, we performed immunoprecipitation of endogenous NOTCH1 (including NICD) in the CLL cell line HG3, including previously published clones with CRISPR/Cas9-engineered heterozygous or homozygous deletion of the FBXW7 WD40 domain [17]. Although the FBXW7/NICD interaction is disrupted by deletion of the WD40 domain, USP28 was co-immunoprecipitated with NOTCH1, suggesting an USP28/NICD interaction mechanism independent of the FBXW7/NICD interaction (Fig. 3C).

**Fig. 4:**
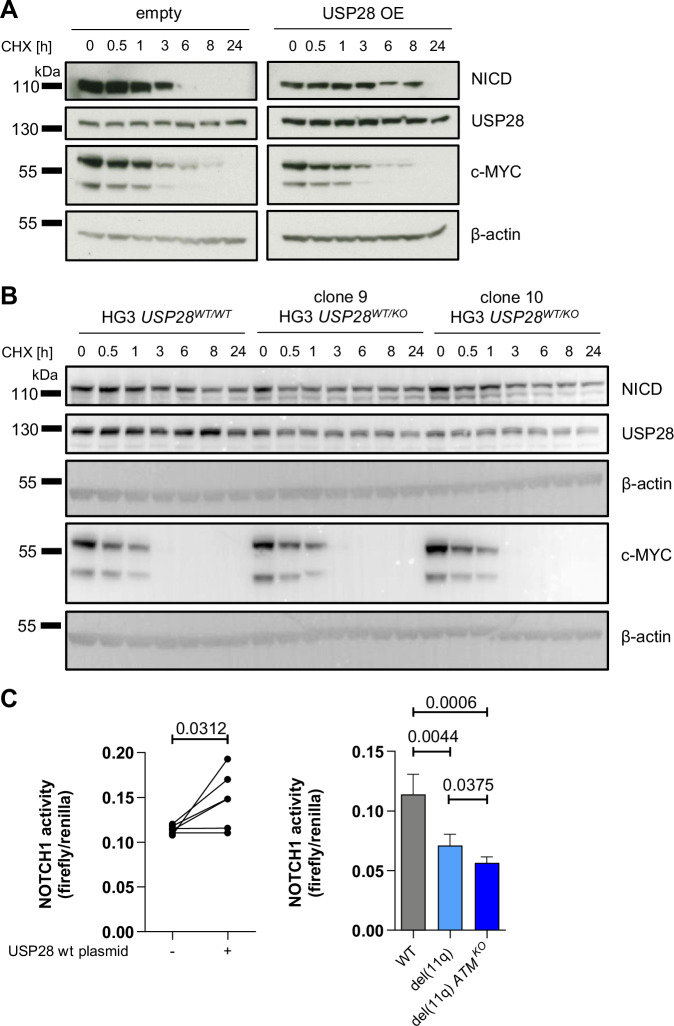
USP28 stabilizes NICD and modulates NOTCH1 signaling activity. NICD protein stability was assessed in (**A**) HEK293 cells with or without overexpression of USP28 (USP28 OE) or in (**B**) HG3 *USP28*^*WT/WT*^ and HG3 *USP28*^*WT/KO*^ cells (clones 9 and 10) after a time course of translational inhibition using cycloheximide (CHX). β-actin was used as loading control. Western blots are representative for three independently performed experiments. **C** NOTCH1 activity measured by luciferase reporter assays (pGL3-Hes1-Luc reporter) in HG3 *USP28*^*WT/WT*^ clones with or without overexpression of a USP28 wt plasmid (*n* = 6 independently performed transfections; left) or in HG3 WT, HG3-del(11q) and HG3-del(11q) *ATM*^*KO*^ cell clones (*n* = 3 clones for each condition; right). Firefly signal was normalized to expression of Renilla luciferase from a constitutively active co-transfected plasmid. Statistical significance was assessed via paired Student’s *t* test (left panel) or one-way ANOVA, followed by Tukey’s multiple comparisons test (right panel). Single data points depict firefly/renilla ratios measured for each single sample and lines connect untransfected with USP28 wt transfected conditions of each repetition (*n* = 6; left). Columns depict mean of firefly/renilla ratios measured for *n* = 3 clones per analyzed cell line and error bars represent standard deviation (right). OE overexpression, wt wild type, WT cell line without modifications.

Next, we investigated whether the interaction with USP28 had consequences for NICD protein stability. For this purpose, we analyzed inhibition of protein translation via cycloheximide (CHX) treatment in HEK293 cells overexpressing USP28 or HG3 cells with CRISPR/Cas9-mediated heterozygous *USP28* knockout reproducing *USP28* knockout similar to del(11q) CLL (*USP28*^*WT/KO*^, Supplementary Fig. 4). Overexpression of USP28 increased stability of NICD compared to cells expressing endogenous levels of USP28 (Fig. 4A). Additionally, NOTCH1 stability was decreased in *USP28*^*WT/KO*^ cells (Fig. 4B) which is in line with previous findings using *USP28* targeting shRNAs [25].

Since regulation of NOTCH1 stability is a crucial mechanism to control NOTCH1 activity we next analyzed whether stabilization of NICD by USP28 overexpression would affect NOTCH1 activity. To test whether the HG3 cell line is a good model for endogenous NOTCH1 activity in CLL we made use of a recombinant human DLL4 Fc chimera protein to stimulate and the γ-secretase inhibitor nirogacestat (Niro) to inhibit NOTCH1 signaling. In HG3 cells, similar to other cell lines, the modulation of NOTCH1 signaling affected NICD and NOTCH1 protein levels and also the viability of HG3 cells was slightly affected suggesting active NOTCH1 signaling in these cells without NOTCH1 pathway mutations [17] (Supplementary Fig. 5). In addition, RNA sequencing and subsequent pathway analysis of HG3 cells treated with DLL4 or nirogacestat revealed significant modulation of the NOTCH pathway (Supplementary Table 2). The overall transcriptional profile of HG3 cells was shown to be comparable to that of the other cell lines tested (Supplementary Fig. 6) suggesting the HG3 cell line is a good model for CLL. Analyzing NOTCH1 activity in *USP28*^*WT/WT*^ HG3 cells using a NOTCH1-responsive luciferase reporter assay, we found that USP28 overexpression increased NOTCH1 activity (Fig. 4C left). Conversely, in HG3 cells with heterozygous *USP28* knockout via CRISPR/Cas9-mediated del(11q), NOTCH1 activity was significantly decreased in comparison to WT cells (Fig. 4C right). These results demonstrate that USP28 overexpression enhances NOTCH1 activity, while heterozygous *USP28* knockout by del(11q) diminishes it.

### USP28 affects NOTCH1 target genes in CLL patients and cell lines

To further measure the effect of USP28 on NOTCH1 activity, we analyzed the expression of NOTCH1 target genes in non-del(11q) patients of the CLL8 cohort [35], stratified by *USP28* expression levels that were higher or lower than the median. For this analysis, we selected NOTCH1 target genes that were experimentally validated to be CLL-specific [14, 15, 17]. In patient samples with low *USP28* expression, 18 out of 19 NOTCH1 target genes were significantly differentially expressed, with 13 genes upregulated and 5 genes downregulated (Supplementary Fig. 7). These findings confirm that USP28 significantly influences NOTCH1 target gene expression.

To investigate how these NOTCH1 target genes are dysregulated in the context of del(11q), we analyzed the expression data from del(11q) CLL patients with deletion of *USP28* from the CLL8 cohort [34, 35]. We observed a significant upregulation of 2 and downregulation of 8 out of 19 NOTCH1 target genes in del(11q) patient samples compared to non-del(11q) patient samples, and a non-significant trend towards downregulation of five additional analyzed genes (Supplementary Fig. 8). Interestingly, in 7 out of 19 genes the statistically significant dysregulation pattern was opposite to that observed in non-del(11q) *USP28* low-expressing patient samples, possibly due to the multifactorial dysregulation in del(11q) patients caused by the simultaneous deletion of *ATM* and other genes. While this highlights the dysregulation of NOTCH1 signaling in del(11q), it cannot be fully explained by the USP28-driven effect.

Next, we aimed to identify genes that are directly bound by the NOTCH1 transcriptional complex and whose expression is modulated by USP28 in the context of del(11q) CLL. Therefore, we performed RNA-Seq of the *USP28*^*WT/KO*^ cell clones and ChIP-Seq targeting RBPJ, the primary transcriptional mediator of NOTCH signaling [38], in the CLL cell line HG3.

The ChIP-Seq targeting RBPJ in HG3 cells identified 12 883 RBPJ binding sites (Supplementary Fig. 9A). The RBPJ binding motif was enriched within the peaks, validating the specificity of the ChIP-Seq (Supplementary Fig. 9B). Moreover, CUT&Tag revealed that the majority of the identified RBPJ peaks were enriched for H3K27ac, suggesting their association with active chromatin (Supplementary Fig. 9A and D).

**Fig. 5:**
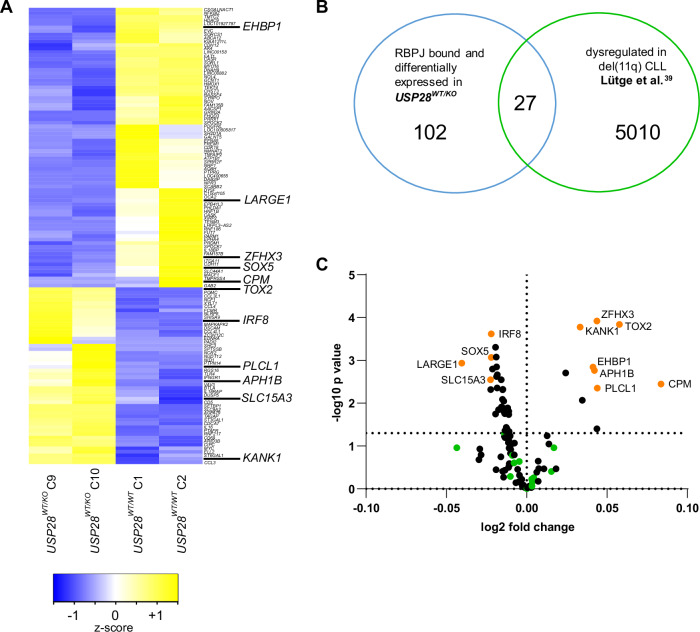
11q deletion and heterozygous loss of *USP28* define a set of 11 dysregulated NOTCH1 target genes. **A** Differential gene expression profiles (RNA-Seq) of the *USP28*^*WT/KO*^ (clones 9 and 10) and *USP28*^*WT/WT*^ cell lines (clones 1 and 2). Gene expression z-scores are color-coded, with blue indicating downregulation and yellow indicating upregulation. The differential expression analysis was performed on a set of pre-selected genes which were identified to be bona fide NOTCH1 targets in an RBPJ ChIP-Seq experiment performed on HG3 wt cells (Supplementary Fig. 9). **B** Venn diagram illustrating the intersection of: the identified RBPJ-bound, differentially expressed genes in *USP28*^*WT/KO*^ (*n* = 129; blue circle) and significantly differentially expressed genes in del(11q) CLL patients from Lütge et al. [39] (*n* = 5037 genes in del(11q) samples differentially expressed, 27 genes overlapping with *USP28*^*WT/KO*^ dysregulated genes; green circle). **C** Volcano plot showing the differential expression of NOTCH1 target genes identified by RBPJ ChIP-Seq in HG3 cells and significantly dysregulated in the *USP28*^*WT/KO*^ cell lines as shown in panel (**A**), in del(11q) CLL patients of the CLL8 cohort [35]. The x-axis represents the log2 fold change of gene expression between patients with del(11q) and patients without del(11q). The y-axis shows the negative log10 of the *p*-value, indicating the significance of the differential expression. The dashed line gives the significance threshold of *p* = 0.05. Genes significantly dysregulated between del(11q) and non-del(11q) patients from Lütge et al. [39] but not in the CLL8 dataset [35] are highlighted in green. The 11 genes marked in orange are significantly differentially expressed between del(11q) and non-del11q) patients in both datasets. C clone.

Subsequent analysis of RBPJ-bound genes in *USP28*^*WT/KO*^ RNA-Seq data revealed 129 genes that were significantly differentially expressed in *USP28*^*WT/KO*^ cells compared to *USP28*^*WT/WT*^ cells (Fig. 5A). To investigate the expression of these genes in del(11q) patients, we intersected the 129 RBPJ-bound and USP28-dependent genes with genes significantly differentially expressed in del(11q) patient samples of the CLL patient cohort published by Lütge et al. [39] and found 27 overlapping genes (Fig. 5B and Supplementary Fig. 10).

Finally, we analyzed the expression of the 129 RBPJ-bound and USP28-dependent genes in del(11q) patients of the CLL8 study cohort [35], overlapping them with the 27 genes shared between *USP28*^*WT/KO*^ cells and Lütge et al. [39] (Fig. 5C).

**Fig. 6:**
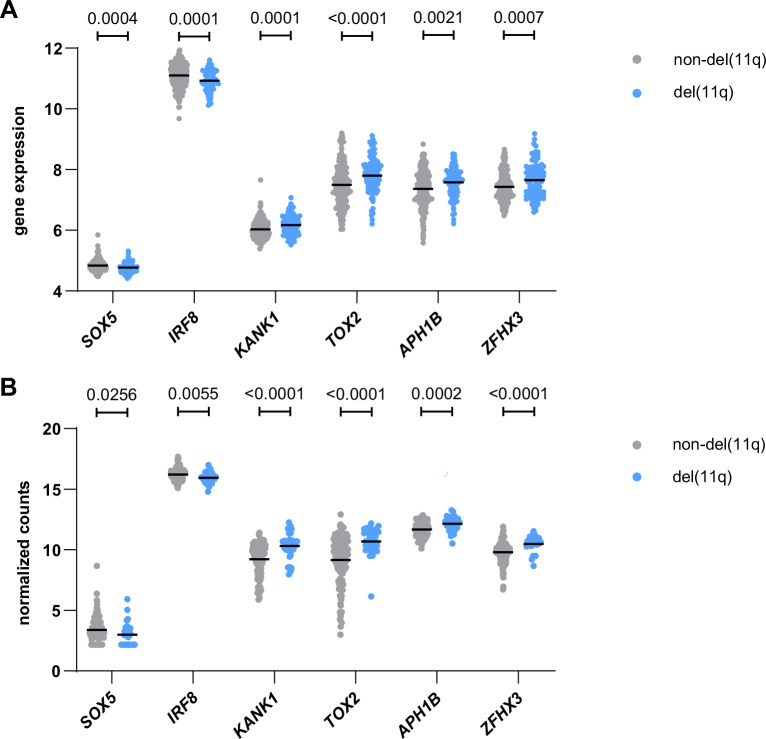
*SOX5, IRF8, KANK1, TOX2, APH1B* and *ZFHX3* are commonly dysregulated in del(11q) CLL. NOTCH1 target gene expression in the data sets of **A** non-del(11q) (*n* = 199) and del(11q) (*n* = 86) CLL patients from the CLL8 gene expression study [35], and **B** non-del(11q) (*n* = 138) and del(11q) (*n* = 31) patients analyzed in Lütge et al. [39]. Statistical significance was assessed via Mann–Whitney test. Lines depict mean, data points represent single patients.

Remarkably, 11 genes (*IRF8, SOX5, LARGE1, SLC15A3, KANK1, TOX2, ZFHX3, EHBP1, APH1B, PLCL1*, and *CPM*) were significantly differentially expressed in all three datasets, (Fig. 5C and 6 and Supplementary Fig. 11). To verify the direct modulation of these genes by downregulation of NOTCH1 signaling, we additionally intersected the 129 RBPJ-bound and USP28-dependent genes and the del(11q) dataset from Lütge et al. [39] with genes significantly changed in HG3 cells upon NOTCH1 inhibition. This analysis showed that 6 genes of the USP28- and del(11q)-dysregulated genes (*CCL3, CCL4, SOX5, KANK1, PRRX1* and *KIAA1211L*) are also affected by pharmacological NOTCH1 inhibition (Supplementary Fig. 12). Combining these results with those from the previous analysis, we define a specific set of 15 NOTCH1 target genes which are significantly affected by downregulation of NOTCH1 signaling due to pharmacological inhibition or heterozygous loss of USP28, including in the context of del(11q). These genes are involved in relevant pathways, aligning with a pathway analysis of the RBPJ-bound genes that are differentially expressed in *USP28*^*WT/KO*^ cells (Supplementary Fig. 9E), and suggesting that they might play a role in del(11q)/USP28-mediated CLL pathogenesis and disease outcome.

### USP28 inhibition is a therapeutic option for CLL

A recent meta-analysis, assessing the impact of BCR or BCL-2 inhibitor treatments on progression free survival (PFS) of refractory and relapsed (R/R) CLL patients, reported that BCR/BCL-2 inhibition increased PFS of del(11q) patients significantly compared to non-del(11q) patients [40]. This suggests a beneficial role of *USP28* deletion via del(11q). Based on this suggestion and the impact of USP28 on NOTCH1 and its target genes in CLL, we next explored the therapeutic potential of targeting USP28 in CLL. To test the feasibility of USP28 as a therapeutic target in vitro and to verify the positive correlation between USP28 and NOTCH1, we used the USP28/25 specific small molecule inhibitor AZ1 [41].

**Fig. 7:**
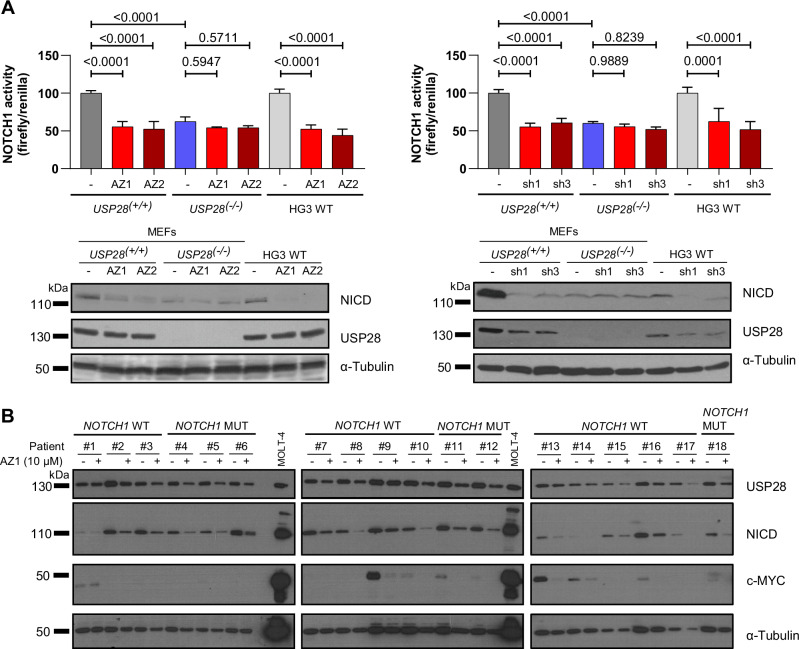
USP28 inhibition decreases NOTCH1 signaling activity in cell lines and NICD protein levels in primary CLL cells. **A** NOTCH1 activity (pGA-981-6(12xCSL) luciferase reporter) and protein levels of NICD and USP28 in cultured mouse embryonic fibroblasts of *USP28*^*(−/−)*^ mice and their respective *USP28*^*(+/+)*^ controls and in HG3 WT cells upon treatment with USP28 inhibitors AZ1 and AZ2 (10 µM each; *n* = 3 for each treatment per cell line; left panel) or knockdown of *USP28* using two different *USP28* shRNA-expressing vectors (sh1 and sh3; *n* = 3 independent transfections per cell line; right panel). α-Tubulin was used as loading control. Statistical significance was assessed via ordinary one-way ANOVA using Šídák’s multiple comparisons test. **B** USP28, NICD and c-MYC protein levels in primary CLL samples (*n* = 18; 12 *NOTCH1* WT and 6 *NOTCH1* MUT) treated with DMSO or the USP28 inhibitor AZ1 (10 µM; 24 h). α-Tubulin was used as loading control and MOLT-4 cell line lysates were used as a positive control. WT wild type, MEFs mouse embryonic fibroblasts, MUT mutated.

In line with the positive correlation between USP28 activity and NOTCH1, treatment of HG3 WT cells and mouse embryonic fibroblasts (MEFs) from *USP28*^*(−/−)*^ and *USP28*^*(+/+)*^ mice with AZ1 and its homolog AZ2 resulted in decreased NOTCH1 activity and NICD protein levels in HG3 WT and *USP28*^*(+/+)*^ MEFs, but not in *USP28*^*(−/−)*^ MEFs (Fig. 7A left). The *USP28*^*(−/−)*^ MEFs generally exhibited decreased NOTCH1 activity, further supporting the positive effect of USP28 on NOTCH1 activity (Fig. 7A). Similarly, specific *USP28* knock down using shRNAs (sh1 and sh3) resulted in reduced NOTCH1 activity (Fig. 7A right). Upon USP28 inhibition using AZ1 in primary CLL cells (*n* = 10; Supplementary Table 3) we observed decreased expression of several NOTCH1 target genes including *FYN, ZMIZ1* and *NRARP* which were previously validated to be CLL specific [14, 15, 17] (Supplementary Fig. 13). In addition, NICD and c-MYC protein levels were significantly downregulated in AZ1 treated cells from CLL patients with and without *NOTCH1/FBXW7* mutations (*NOTCH1/FBXW7* mut *n* = 6, *NOTCH1* wt *n* = 12; Supplementary Table 4; Fig. 7B and Supplementary Fig. 14). These findings conclusively demonstrate that USP28 activity directly influences NOTCH1 signaling and target gene expression.

**Fig. 8:**
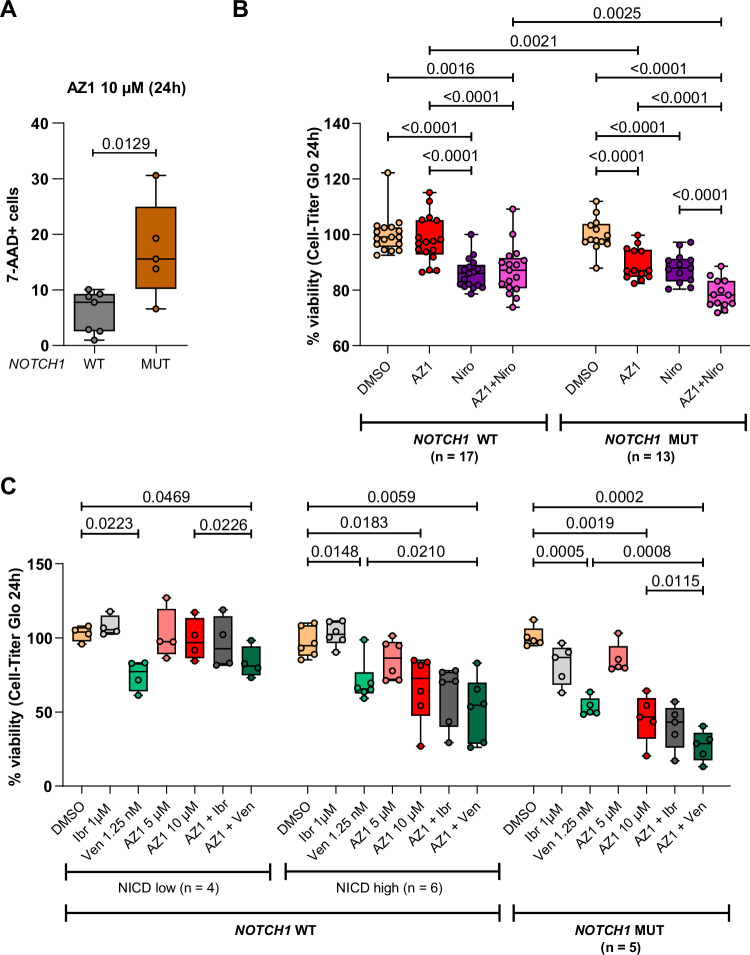
USP28 inhibition decreases CLL cell viability and shows additive effect to venetoclax treatment. **A** Cell viability analysis by 7-AAD staining of primary CLL cells (*n* = 12; 7 *NOTCH1* WT and 5 *NOTCH1* MUT) treated with 10 µM AZ1. Non-viable cells are quantified as the percentage of 7-AAD+ cells in each individual sample. Statistical significance was assessed via unpaired Student’s *t* test. **B** Cell viability analysis by ATP quantification (Cell-Titer Glo) of primary CLL cells (*n* = 30; 17 *NOTCH1* WT and 13 *NOTCH1* MUT; Supplementary Table 5) treated with DMSO, 10 µM AZ1, 1 µM nirogacestat (Niro) or the combination of AZ1 and nirogacestat for 24 h. Lines depict median and boxes the interquartile range, data points represent single patient samples. Statistical significance was assessed via two-way ANOVA, followed by Tukey’s multiple comparisons test. **C** Cell viability analysis by ATP quantification (Cell-Titer Glo) of primary CLL cells (*n* = 15; 10 *NOTCH1* WT (4 NICD low, 6 NICD high) and 5 *NOTCH1* MUT; Supplementary Table 4) treated with 1 µM ibrutinib (Ibr), 1.25 nm venetoclax (Ven), increasing doses of AZ1 (5 and 10 µM) or the combination of 10 µM AZ1 with ibrutinib or venetoclax for 24 h. Lines depict median and boxes the interquartile range, data points represent single patient samples. Statistical significance was assessed via one-way ANOVA, followed by Tukey’s multiple comparisons test. WT wild type, MUT mutated, Niro nirogacestat, Ibr ibrutinib, Ven venetoclax.

Moreover, AZ1 treatment significantly induced cell death and reduced viability in a dose-dependent manner, especially in CLL cells harboring *NOTCH1* mutations (Fig. 8). AZ1 reduced their viability as strongly as the FDA-approved γ-secretase inhibitor nirogacestat highlighting the potential of USP28 inhibition (Fig. 8B; Supplementary Table 5). Finally, we explored the potential of combining AZ1 with venetoclax, a BCL-2 inhibitor, or ibrutinib, a BTK inhibitor, which are both commonly used in the treatment of CLL patients. These drugs show significant clinical activity in CLL but are not uniformly effective, with lower efficacy particularly in patients with *NOTCH1* mutations or activated NOTCH1 signaling [42, 43]. Consequently, we analyzed the effects of combinations of AZ1 with venetoclax or ibrutinib on CLL patient cells with *NOTCH1* mutations or activated NOTCH1 signaling defined by high NICD protein expression and compared them to the effects on CLL cells without *NOTCH1* mutations and low NICD levels. The combination of AZ1 with venetoclax reduced CLL cell viability more efficiently than either treatment alone, suggesting a beneficial effect of dual inhibition of USP28 and BCL-2. In contrast, combining AZ1 with ibrutinib did not lead to an additional reduction of CLL cell viability (Fig. 8C). The combined effect of AZ1 and venetoclax was most pronounced in primary CLL cells with high expression of NICD and cells with mutations in *NOTCH1* (Fig. 8C; Supplementary Table 4). Therefore, we propose that inhibiting USP28 with AZ1 could potentiate the therapeutic effect of venetoclax offering a promising treatment option for patients which typically show inferior responses to conventional treatments.

## Discussion

The heterogeneous character of CLL and the occurrence of treatment refractory and progressive disease make it imperative to find molecular defects that are common across patient groups to 1) develop new effective treatment strategies and 2) prevent refractory and relapsing disease.

Active NOTCH1 signaling and *NOTCH1* mutations are central pathogenic factors in CLL correlated with decreased survival and refractory disease. Importantly, only 5-10% of CLL cases with active NOTCH1 signaling without *NOTCH1* mutations could so far be explained by alternative causes like *FBXW7* mutations [14, 44]. Here we present evidence that USP28-mediated dysregulation of NOTCH1 activity is another cause of activation of NOTCH1 signaling in CLL and propose that targeting USP28 might be of benefit in CLL patients with active NOTCH1 signaling.

### USP28 regulates NOTCH1 signaling in CLL in an oncogenic manner

We found that USP28 knockdown or pharmacological inhibition downregulates NOTCH1 signaling in CRISPR/Cas9-modified HG3 and primary CLL cells, indicating an oncogenic driver function for USP28 in CLL. This aligns with findings from solid cancers such as lung squamous cell carcinoma, colorectal cancer, breast cancer and glioblastoma [25–27, 45–47].

From other cancers it is also reported that besides USP28, the deubiquitinases USP7, USP8, USP10 and USP11 interact with NICD and regulate NOTCH1 signaling [48–57]. In CLL, these deubiquitinases might affect NOTCH1 signaling, as they are expressed at similar or higher levels than USP28 (Supplementary Fig. 15). While USP7 and USP11 have broad roles in the regulation of DNA-damage response promoting CLL and T-ALL cell survival, their specific impact on NOTCH1 signaling might be less significant [48–51, 56, 57]. USP10 has been reported to play a role in AML pathogenesis but not in connection with NOTCH1 signaling [55]. For USP8 so far, no role in hematological malignancies has been reported. Although the evidence for the functional interaction of USP8 and USP10 with NOTCH1 signaling is clearly shown, their cytoplasmic localization potentially limits their direct impact on NOTCH1 transcriptional activity. Also, the USP28 homolog USP25 is located in the cytoplasm but targets different proteins than USP28. Therefore, USP25 might be less relevant for NOTCH1 signaling [31]. In contrast, USP28 is localized in the nucleus and might therefore have immediate and specific impact on NOTCH1 signaling activity in CLL cells. This specific impact of USP28 on NOTCH1 signaling is supported by the findings of our study showing downregulation of NOTCH1 signaling with USP28 deletion or inhibition and underlined by the effect of USP28 inhibition on CLL cell viability.

The hypothesis of USP28 as an oncogenic regulator in CLL is additionally supported by our observation that NOTCH1 signaling is not upregulated in CLL cells with del(11q) as it would have been expected in a simplistic model of the tumor suppressor mechanism in 11q. Our findings are in line with previous reports that NICD protein levels are the lowest in del(11q) [58]. However, elucidation of the exact mechanism of pathogenesis in del(11q) CLL is still ongoing. Currently, del(11q) and loss of *BIRC3* have been postulated to activate non-canonical NF-κB signaling leading to increased BCL-2 [59]. Other hypotheses involve a defective DNA-damage response due to *ATM* loss where USP28 could be involved due to its first described role in 53BP1 stabilization [31, 36].

Furthermore, USP28 is involved in the regulation of additional genes that are relevant for CLL pathogenesis like c-MYC and HIF-1α [22, 29]. And indeed, upon USP28 inhibition we observed downregulation of c-MYC protein levels in primary CLL cells, which suggests that USP28 mediates modulation of c-MYC in primary CLL cells as well.

In contrast, there are reports about USP28 acting as a tumor suppressor [28, 60], and USP28 affecting FBXW7-autoubiquitination causing FBXW7 stabilization which results in degradation of target proteins [61]. This suggests that the function of USP28 might be context-dependent and likely involves additional factors which add an additional layer of regulation.

### USP28 interacts with NICD independently of FBXW7

Here we show that USP28 interacts with FBXW7 independently of the FBXW7 WD40 domain which confirms previous findings [22]. Additionally, we found that USP28 does not require intact FBXW7 to interact with NICD suggesting a similar mechanism as recently proposed for the interaction of USP28 with c-MYC [26]. This mechanism includes that USP28 interacts with the unphosphorylated CPD of c-MYC in the absence of FBXW7 but when FBXW7 is present USP28 can only bind the phosphorylated CPD in cooperation with FBXW7 [26]. So far, we could not test whether the phosphorylation status of the CPD in the NICD PEST domain regulates the binding preference for only USP28 or USP28 together with FBXW7. However, since we observed that USP28 could interact with NICD variants with almost complete deletion of the PEST domain it might be possible that phosphorylation of the CPD does not play a critical role in the USP28/NICD interaction. Interestingly, this suggested PEST-independent interaction mechanism might explain the observation of USP28 inhibition reducing NICD levels in CLL patients with PEST-deleting *NOTCH1* mutations, but raises questions about other domains of the NOTCH1 protein that might facilitate the USP28/NICD interaction and NOTCH1 stabilization in CLL.

### 11q deletion and heterozygous loss of *USP28* define a set of 11 dysregulated NOTCH target genes

Our analysis of pre-selected classical NOTCH1 target genes such as *HEY* and *HES* gene families in CLL patients comparing high/low *USP28* expressing patient samples or del(11q) with non-del(11q) patient samples showed a clear dysregulation of NOTCH1 signaling. Unexpectedly, most of these genes were upregulated in *USP28* low-expressing patient samples and in addition anticorrelated with del(11q) patients. These observations might be explained by context-specific regulation of NOTCH1 target genes due to 1) multifactorial dysregulation in del(11q) patients via deletion of several genes on 11q affecting e.g. epigenetic mechanisms regulating NOTCH1 target genes or 2) different levels of *USP28* expression in *USP28* low-expressing samples compared to del(11q) samples. To identify additional USP28-dependent NOTCH1 target genes, we combined HG3 WT ChIP-Seq data with expression data from HG3 *USP28*^*WT*/*KO*^ cell clones and CLL patients with and without del(11q) from two different datasets [35, 39]. In addition, we intersected the cell line gene expression data and one of the del(11q) patient datasets with gene expression in HG3 cells upon NOTCH1 inhibition. This approach revealed 15 NOTCH1 target genes specifically dysregulated by NOTCH1 and USP28 in CLL with and without del(11q).

Among these genes, *IRF8* and *TOX2* encode for transcription factors involved in immune cell regulation and development, with IRF8 playing a tumor suppressor role in AML [62] and TOX2 being involved in regulation of T-cell response in CLL [63]. SOX5, KANK1, and ZFHX3 are transcription factors implicated in specific cancer entities and regulate cell development, migration, and genomic stability. They interact with signaling pathways such as RUNX3, CDKN1A, and HIF-1α [64–69]. APH1B is a subunit of the γ-secretase complex required for NOTCH1 cleavage and directly impacts NOTCH1 signaling [70]. CCL3 and CCL4 are chemokines contributing to the remodeling of the CLL microenvironment, are important biomarkers for BCR activation and correlate with poor prognostic markers for CLL progression [71, 72].

These USP28-dependent NOTCH1 target genes belong to important pathways and could play roles in del(11q)/USP28-mediated CLL pathogenesis. Further investigation into their specific functions may provide valuable insights into disease progression and potential therapeutic targets.

### USP28 inhibition with AZ1 is a new treatment strategy to inhibit NOTCH1 signaling in CLL

Interestingly, it has been recently reported that R/R del(11q) patients benefit more from BCR/BCL-2 inhibition in terms of improved PFS than patients without del(11q). This clinical data supports our hypothesis of USP28 as an oncogenic factor affected by del(11q), and that CLL patients might benefit from USP28 inhibition. In addition, USP28 inhibition with AZ1 has already been found to be an effective therapeutic option in lung or breast cancer [27, 37, 46, 73–75]. AZ1 treatment in CLL cell lines and in primary CLL cells reduced NOTCH1 signaling and CLL cell viability, suggesting a similar therapeutic effect in CLL. Especially in *NOTCH1* mutated patient cells the effect of AZ1 was as strong as the γ-secretase inhibitor nirogacestat indicating that USP28 inhibition is a relevant therapeutic option for this group of patients. The combination of AZ1 with venetoclax showed additive effects on CLL cell viability, which was especially observed in cells from patients with high NICD levels or *NOTCH1* mutations. Interestingly, *NOTCH1* mutations in CLL are correlated with low BAX/BCL-2 ratios [76] and a recent study suggested that NOTCH1 activation provides growth advantage to CLL cells to potentially escape venetoclax induced apoptosis [77]. Additionally, in prostate cancer it has been shown that *NOTCH1* silencing decreased BCL-2 levels which increased sensitivity to chemotherapy [78], suggesting a similar mechanism for the interplay of AZ1 and venetoclax. So far, venetoclax is commonly combined with anti-CD20 antibodies to obtain optimal CLL treatment outcomes [79, 80]. However, *NOTCH1*-mutated patients do not benefit from anti-CD20 therapy [8, 9, 81]. Therefore, especially for CLL patients with active NOTCH1 signaling, AZ1 or other USP28 inhibitors such as CT1113 recently tested in T-ALL [82] might be optimal partners for rational therapeutic combinations with venetoclax.

Given that active NOTCH1 signaling in CLL predicts poor outcomes and is difficult to target [5–9, 21], it is crucial to identify new therapeutic targets to provide effective treatment options for these patients. Here we show that USP28 dysregulates NOTCH1 signaling in CLL and demonstrate that USP28 inhibition with the small molecule inhibitor AZ1 could be a new therapeutic option in CLL (Fig. 8). We therefore propose that USP28 serves as an additional dysregulatory layer of NOTCH1 signaling in CLL cells, presenting a new Achilles heel for CLL.

## Supplementary information

Supplemental material file

## Data Availability

RNA-Seq data and ChIP-Seq data are openly available at the gene expression omnibus platform (GEO) under the accession numbers GSE229756 (RNA-Seq *USP28*^*WT/KO*^ cell lines), GSE293100 (RNA-Seq DLL4/Nirogacestat treated cell lines) and GSE275036 (ChIP-Seq). Researchers are encouraged to refer to the supplemental materials for detailed methods and data processing information. Any further inquiries regarding the data can be directed via e-mail to the corresponding authors.
