# Supplementary material for: NOTCH1 signaling is dysregulated by loss of the deubiquitinase USP28 with del(11q), uncovering USP28 inhibition as novel therapeutic target in CLL
Source: Leukemia. 2025 Jun 2;39(8):1892–904. doi: 10.1038/s41375-025-02632-4 (PMC12310537; doi:10.1038/s41375-025-02632-4)
Supplement: Supplementary file 1 — Supplemental material file [file 41375_2025_2632_MOESM1_ESM.pdf]

NOTCH1 signaling is dysregulated by loss of the deubiquitinase USP28 with del(11q), uncovering USP28 inhibition as novel therapeutic target in CLL

Alena Sophie Ehrmann[1,2]*, Miguel Quijada-Álamo[3,4]*, Viola Close[1]*, Min Guo[5]*, Valentina Carracoi[1], Claudia Pérez-Carretero[3], Luis Antonio Corchete[3], Tobias Friedrich[6], Benedetto Daniele Giaimo[6], Deyan Yordanov Yosifov[1,2], Johannes Bloehdorn[1], Alberto Rodríguez-Sánchez[3], Eugen Tausch[1], Christof Schneider[1], Hartmut Döhner[1], Thomas Kietzmann[7], Tilman Borggrefe[6], Stephan Stilgenbauer[1], Franz Oswald[5,8§], Jesús-María Hernández-Rivas[3§] and Daniel Mertens[1,2,8§]

[1]University Hospital Ulm, Department of Internal Medicine III, Albert-Einstein-Allee 23, 89081 Ulm, Germany.

[2]German Cancer Research Center (DKFZ), Bridging Group Mechanisms of Leukemogenesis, B061, Im Neuenheimer Feld 280, 69120 Heidelberg, Germany.

[3]University of Salamanca, Department of Medicine, University Hospital of Salamanca, IBSAL, IBMCC, CSIC, Cancer Research Center, Campus Miguel de Unamuno s/n, 37007, Salamanca, Spain.

[4]Icahn School of Medicine at Mount Sinai, Department of Oncological Sciences, New York, 10029, NY, USA.

[5]University Hospital Ulm, Department of Internal Medicine I, Albert-Einstein-Allee 23, 89081 Ulm, Germany.

[6]Justus-Liebig-University Giessen, Institute of Biochemistry, Friedrichstrasse 24, 35392 Giessen, Germany.

[7]University of Oulu, Faculty of Biochemistry and Molecular Medicine and Biocenter Oulu, Aapistie 7C, 90014, Oulu, Finland

[8]Corresponding authors: Franz Oswald, e-mail: franz.oswald@uni-ulm.de; Jesús-María Hernández-Rivas, e-mail: jmhr@usal.es; Daniel Mertens, e-mail: d.mertens@dkfz-heidelberg.de

*Equal contribution - first author

[§]Equal contribution - senior author

Competing interests statement: The authors declare no competing financial interests in relation to this work.

Supplemental Material

Cell culture conditions

Cell lines were incubated at 37°C and normoxia with 5% CO_2. The CLL cell line HG3 (DSMZ: ACC765), all HG3-derived clones and PGA-1 (ACC766), Granta-519 (ACC342), MEC-1 (ACC497) and MO1043[1] were cultured in 90% RPMI 1640 (Sigma-Aldrich, St. Louis, MD, USA) + 10% heat inactivated (h.i.) FBS (Biochrom, Berlin, Germany). HEK293 (DSMZ: ACC305) and HeLa (ATCC: CCL2) cells were cultured in 90% Dulbecco's MEM (Gibco, Grand Island, NY, USA; with 25 mM HEPES) + 10% h.i. FBS (Biochrom).

Mouse embryonic fibroblasts (MEFs) of *USP28*$^{(-/-)}$ and *USP28*$^{(+/+)}$ mice[2] were isolated from embryos at embryonic day 10.5 according to the protocol of Durkin and colleagues[3]. MEFs were cultured in Dulbecco's modified Eagle's medium (Sigma-Aldrich) supplemented with 10% fetal bovine serum (Biowest, Nuaillé, France), 1% nonessential amino acids (Sigma-Aldrich), and 1% antibiotic in a standard cell culture incubator. MEFs were plated in 6-cm^2 plates (5×10^5 cells/plate) and cultured in normoxia for 24 h. At the time of harvest the plates were 85% confluent.

Generation of HG3 CRISPR/Cas9 cell lines

A candidate single-guide RNA (sgRNA) targeting *USP28* exon 4 was designed using CRISPick (https://portals.broadinstitute.org/gppx/crispick/public) and selected based on its predicted on-target efficiency and the lowest off-target effects (5' CTCCAGTAGACTCAAAGCAA 3'). Moreover, a sgRNA sequence designed not to target the human genome was used as a negative control (5' ACGGAGGCTAAGCGTCGCAA 3'). sgRNAs were cloned into the pLKO5.sgRNA.EFS.GFP vector (Addgene_#57822) and transfected into HG3-Cas9 using the Amaxa Nucleofector II system (Lonza, Basel, Switzerland; program T-016). 48 hours post-transfection, GFP^+ cells were sorted using a FACSAria II flow cytometer (BD Biosciences, Franklin Lakes, NJ, USA) and single-cell seeded in 96-well plates. Single-cell clones were subsequently expanded and screened for the presence of truncating mutations in target sequences using Sanger sequencing and western blotting.

Treatment compounds

Cycloheximide (CHX; Sigma-Aldrich, Steinheim, Germany) diluted in H_2O was used in HG3 and HEK293 cells at 20 µg/ml or 100 µg/ml, respectively and incubated for the indicated time course to assess protein stability upon translation inhibition.

For USP28 inhibition 10 µM of AZ1 or AZ2 (Selleckchem, Cologne, Germany or AstraZeneca, Cambridge, UK) diluted in DMSO (Sigma) was used in all cell lines and primary cells for 24 hours. Treatment of primary CLL cells with venetoclax (Selleckchem) diluted in DMSO was carried out at 1.25 nM and ibrutinib (LC laboratories, Woburn, MA, USA) diluted in DMSO was used at a concentration of 1 µM for 24 hours.

To stimulate NOTCH1 signaling in cell lines with the soluble NOTCH1 ligand DLL4, cells were incubated for 24 hours with recombinant Human DLL4 Fc Chimera Protein (R&D Systems, Minneapolis, MN, USA), reconstituted in phosphate buffered saline (PBS) and diluted to 4 µg/ml, as previously described[4].

Inhibition of NOTCH1 signaling with the γ-secretase inhibitor nirogacestat (Hycultec, Beutelsbach, Germany) diluted in DMSO was carried out at 1 µM for 24 hours.

Plasmids, transfection and luciferase assay

USP28 phosphorylation sites (serine 67 and serine 714) were derived from Zhang et al.[5]. Commercially synthesized sequences of the variant specific parts were cloned into the USP28 wt construct which was purchased as pDZ USP28 Flag from AddGene (plasmid #15665) and cloned into pcDNA3 vector backbone. The GFP-USP28 fusion constructs were cloned by exchange of the amino-terminal Flag-Tag with the GFP cDNA as HinDIII fragment. The GFP-FBXW7α expression vector and the wildtype or mutant Flag-FBXW7 variants were described previously[6]. The NICD wt plasmid was constructed by cloning the cDNA encoding wild type NICD, starting at amino acid 1761 of human NOTCH1 (reference NP_060087) till the last amino acid 2555, into pcDNA3.1 vector backbone. The USP28 shRNAs sh1 and sh3 were published previously in Flügel et al.[7] and the NOTCH1-specific firefly-luciferase reporters pGA-981-6(12xCSL) and pGL3-Hes1-Luc were already described by Oswald et al.[8].

The pSV-Renilla plasmid was purchased from Promega and co-transfected with the luciferase reporters for internal normalization purposes. Cells were seeded 24 hours prior to transfection in the corresponding medium. HEK293 cells were transfected using the calcium phosphate mammalian transfection kit (Promega, Madison, WI, USA) and CLL cell lines were transfected via electroporation using the Nucleofector II device (Lonza) and Cell line Nucleofector Kit T (Lonza) according to the manufacturer's instructions. Cells were lysed 24 hours after transfection and after addition of the Dual-Luciferase reporter assay system buffers (Promega) luminescence of the firefly and renilla luciferases were measured using a Glomax Discover microplate reader (Promega). NOTCH1-reporter luminescence (firefly luciferase) was normalized to luminescence of the renilla control luciferase.

Immunofluorescence microscopy

HeLa cells were cultured on chamber slides (#155380, Thermo Scientific Nunc, Rockford, IL, USA) treated with 0.1 mg/ml fibronectin (Roche, Mannheim, Germany) at a density of 10^5 cells per cm^2. After 16 hours, cells were transfected with 250 ng of expression plasmids indicated in the corresponding figure legend using the Nanofectin transfection reagent (PAA, Pasching, Austria). Cells were washed with PBS 24 hours after transfection, fixed with 4% PFA (in PBS, pH 7.5) and permeabilized with 0.1% Triton X-100. After blocking with 1 ml blocking buffer (0.2% gelatine from cold water fish skin (Sigma-Aldrich) for 30 min at RT cells were incubated with the primary antibody against Notch1 (ab128076, Abcam, Cambridge, UK) and the secondary antibody goat anti-mouse polyclonal Alexa Fluor 568 (Invitrogen, Carlsbad, CA, USA)]. Cells were then stained with DAPI [2-(4-carbamimidoylphenyl)-1H-indol-6-carboximidamide (Merck, Darmstadt, Germany)], embedded in Fluoromount-G (#0100-01, SouthernBiotec, Birmingham, AL, USA) and stored at 4°C overnight. Imaging was performed with an IX71 fluorescence microscope (Olympus, Tokyo, Japan) equipped with a digital camera (C4742, Hamamatsu Photonics, Hamamatsu, Japan), and a 100-W mercury lamp (HBO 103W/2, Osram, Munich, Germany). The following filter sets were used: Green (GFP), ex: HQ470/40, em: HQ525/50; Red (Alexa-Flour-568), ex: HQ545/30, em: HQ610/75; Blue (DAPI), ex: D360/50, em: D460/50.

Immunoprecipitations

Cells (HEK293) were transfected with the indicated constructs for expression of NICD, USP28 and FBXW7 protein variants with or without Flag-tag. 24 h after transfection, cells were lysed in 600 µl CHAPS lysis buffer [10 mM 3-[(3-Cholamidopropyl)-dimethylammonio]-1-propanesulfonate hydrate (CHAPS, Merck), 50 mM Tris-HCl (pH 7.8), 150 mM NaCl, 5 mM NaF, 0.5 mM Phenylmethanesulfonyl fluoride (PMSF, Merck) and 40 µl/ml cOmplete protease inhibitor cocktail (Roche)]. Extracts were incubated with agarose-conjugated anti-Flag antibody (M2, Merck) overnight at 4 °C. After six washing steps with CHAPS lysis buffer, the precipitates were resuspended in 1x SDS-polyacrylamide gel loading buffer. NICD, USP28, FBXW7 and co-immunoprecipitated proteins were analyzed by Western blotting using the following antibodies: anti-Notch1 (#3447S, cell signaling technology), anti-USP28 (HPA006778, Sigma-Aldrich), anti-FBXW7 (28424-1-AP, Proteintech, Rosemont, IL, USA) and anti-FLAG (F4042, Sigma-Aldrich). Anti-α-Tubulin (T9026, Sigma-Aldrich)was used as expression control.

To immunoprecipitate endogenous NOTCH1 (including NICD) in HG3 cells, cells were lysed in 600 µl of CHAPS lysis buffer supplemented as indicated above. The cell extracts were incubated with an anti-Notch1 antibody (#3447S, cell signaling technology) or an isotype control antibody (rat IgG2b, 400601, BioLegend, San Diego, CA, USA) overnight at 4 °C. Afterwards, this solution was incubated with protein A agarose beads (#9863, cell signaling technology) for two hours at 4 °C. Subsequently, the beads were washed three times with CHAPS lysis buffer, resuspended in 1x SDS-polyacrylamide gel loading buffer and analyzed by Western blotting.

Western Blotting

Cells were lysed for 10 minutes on ice with RIPA buffer (Sigma-Aldrich) supplemented with Complete™ EDTA-free protease inhibitor cocktail (Roche) and PhosSTOP™ (Roche). After 15 minutes of centrifugation at 4 °C and 13.000 rpm the supernatant was collected. Protein concentration of the lysates was obtained using the Pierce™ BCA Protein Assay kit (Thermo Scientific, Rockford, IL, USA). The samples were mixed with 4x NuPage LDS sample buffer (Invitrogen) and boiled at 95 °C for 5 minutes. Next, samples were loaded onto 4-12% NuPage

Bis-Tris gels (Invitrogen) and electrophoresis was run at 200 V for 50 minutes in 1x MOPS buffer (Invitrogen). Proteins were transferred by semi-dry blotting with 1x NuPage Transfer buffer (Invitrogen) to PVDF-membranes (Thermo Scientific). The following primary antibodies were used to analyze protein expression: USP28 (HPA006778, Sigma-Aldrich), NOTCH1 (#3608, cell signaling technology, Danvers, MA, USA), NICD (cleaved Notch1 (Val1744), #4147, cell signaling technology), c-MYC (ab32072, Abcam or #13987, cell signaling technology), Cyclin-E (sc-481, Santa Cruz, Dallas, TX, USA), c-JUN (sc-44, Santa Cruz) and FBXW7 (28424-1-AP, Proteintech). As loading controls, we used: β-actin (#8457, cell signaling technology or A3854, Sigma-Aldrich) or α-tubulin (#2146, cell signaling technology). The secondary antibodies were purchased from Dako (Hamburg, Germany). Densitometric quantification was performed with Fiji ImageJ[9] and protein levels calculated relative to the loading control.

Quantitative real-time PCR

Total RNA was isolated from primary CLL cells using the mRNeasy Mini Kit (Qiagen, Hilden, Germany). DNA was digested using DNAse I (Thermo Scientific) and subsequent reverse transcription was carried out using the LunaScript RT SuperMix kit (New England Biolabs, Frankfurt am Main, Germany). Luna Universal Probe qPCR Master Mix (New England Biolabs) was used to set up the qPCR reaction in an Applied Biosystems QuantStudio device (Thermo Scientific). Expression of the following genes was analyzed using the LinRegPCR software[10] and normalized to the expression of three housekeeping genes HPRT, PPIA and TBP.

QuantiTect Primer Assays from Qiagen: NOTCH1 (QT01005109), FYN (QT00054005), ZMIZ1 (QT00036925), NRARP (QT00249109). Housekeeping genes from Qiagen: HPRT (QT00059066), PPIA (QT00052311), TBP (QT00000721).

RNA-Seq

The cell lines HG3, PGA-1, Granta-519, MEC1 and MO1043 were treated in duplicates as described above with soluble DLL4 or nirogacestat. RNA sequencing and data analysis were performed by Biomarker Technologies (BMK) GmbH (Münster, Germany). Total RNA from the

different cell lines (HG3, PGA-1, Granta-519, MEC-1 and MO1043) was extracted using the mRNeasy Mini Kit (Qiagen) and quality control was performed using NanoDrop, Qubit 2.0, and Agilent 2100. Libraries were constructed using mRNA isolated by poly-T oligo-attached magnetic beads followed by random fragmentation and cDNA synthesis with random hexamers. Double-stranded cDNAs were end-repaired, adenylated, and ligated to adapters before PCR amplification. After quality control, sequencing was performed on an Illumina Nova PE150 platform. Clean data with high quality were obtained by filtering raw data to remove adapter sequences and low-quality reads. The clean reads were mapped to the human reference genome (Homo_sapiens.GRCh38_release95.genome.fa) using HISAT2. Mapping statistics showed high mapping ratios ranging from 94.27% to 99.25%. StringTie was used for transcript assembly and gene expression quantification in Fragments Per Kilobase of transcript per Million fragments mapped (FPKM). Differential expression analysis between treatment conditions (DLL4 stimulation or nirogacestat treatment vs. control) was performed using edgeR with a threshold of Fold Change ≥ 1.5 and p-value < 0.05. Gene Set Enrichment Analysis (GSEA) was performed without setting thresholds on fold change to detect weak alterations in gene expression within specific pathways. Principal component analysis was performed using prcomp in R and visualized using the factoextra package.

HG3^{WT} (n=2) and HG3 *USP28*$^{WT/KO}$ (n=2) clones were analyzed by RNA-Seq following the TruSeq Stranded mRNA protocol (Illumina, San Diego, CA, USA). Briefly, the RNA library was prepared using 200 ng of total RNA from each sample and mRNAs were purified using poly-T oligo-attached magnetic beads and subsequently followed a fragmentation step. mRNA fragments were used as templates for first-strand cDNA synthesis by reverse transcription with random hexamers. Upon second-strand cDNA synthesis, double-stranded cDNAs were end-repaired and adenylated at the 3′ ends. Universal adapters were ligated to the cDNA fragments, then the sequencing library of DNA fragments that had adapters on both ends was amplified by PCR and used to produce the clusters that were then sequenced in a NextSeq 550 platform (Illumina). Each sample was sequenced in a separate flow cell lane, producing 24.6 - 35.7 million paired-end reads per sample, with a final length of 76 bases. The quality

information of the resulting FASTQ files was checked using the FastQC (v0.11.9) [Andrews S. (2010). FastQC: a quality control tool for high throughput sequence data. Available online at: http://www.bioinformatics.babraham.ac.uk/projects/fastqc] and MultiQC (v1.13) tools. Paired-reads were mapped against the GRCh37 version of the human genome using the STAR aligner (v2.7.10a)[11] and gene annotations from Ensembl (v85). Gene level counts were obtained from the BAM files through the HTSeq package (v0.11.2)[12] in its "union" mode. Low expressed genes across samples were removed so that only those genes with 5 or more assigned reads in at least two samples were considered for subsequent analyses. The resulting count matrix was normalized to the estimated size factors using the DESeq2 (v1.36.0)[13] median of ratios method. A classical multidimensional scaling (MDS) was used to visualize the relationship among the analyzed samples. This MDS was performed considering the Euclidean distance as the distance measure and the group average as the linkage method. Graphical representation of this MDS was made using the ggplot2 package (v3.4.0)[14] in R (v4.2.1). Differential gene expression analysis was performed using a Wald test implemented in the DESeq2 R package. Genes with a Benjamini-Hochberg adjusted *p*-value (FDR) < 0.05 were considered significant.

For the integrative analysis to identify RBPJ-bound genes (see ChIP-Seq below) differentially expressed in the HG3^{WT} (n=2) and HG3 *USP28*$^{WT/KO}$ (n=2), the RNA-Seq data were processed as follows. Sequencing data underwent quality and adapter trimming using TrimGalore v.0.6.5 (https://github.com/FelixKrueger/TrimGalore). The resulting trimmed reads were aligned to the human genome (hg19) and stored as BAM files. This alignment was performed using HISAT2 v.2.2.[15] with parameters "–k 1 –min-intron 30 –max-intron 3000". The gene-annotation-counts table, encompassing all samples, was generated using the summarizedOverlaps function from the GenomicAlignments R/BioConductor package[16] using the corresponding Gene Transfer Format file (Illumina's IGenomes). Normalization and subsequent identification of differentially expressed genes (log2FC > 0.05 or < -0.05 and FDR < 0.05) were conducted using DESeq2 v.1.28.1[13]. Analysis of significantly enriched pathways was performed using Metascape[17].

ChIP-Seq

Chromatin immunoprecipitation (ChIP) was performed as previously described[18] using an anti-RBPJ antibody (Cell Signaling Technology, 5313S). Chromatin from *Drosophila melanogaster* Schneider cells was used for spike-in purposes and immunoprecipitated with 2 µg of anti-His2Av antibody (Active Motif 61686). Libraries were prepared using the Diagenode MicroPlex Library Preparation kit v3 (Diagenode C05010001) following manufacturer's instructions with few modifications. Libraries were purified with Agencourt AMPure XP Beads (Beckman Coulter, #A63881), quantified and analyzed on an Agilent Tapestation device. Finally, sequencing was performed on a NovaSeq device at Novogene UK.

Raw sequencing reads were adapter and quality trimmed using TrimGalore v.0.6.5. Trimmed sequencing reads were aligned against the human reference genome (hg19 from UCSC) using HISAT2 v.2.2.1 with "--no-spliced-alignment" parameter and stored as BAM files. Subsequently, BAM files were filtered for PCR duplicates using Picard tools (https://broadinstitute.github.io/picard/). Peak calling for RBPJ binding sites was performed using MACS2 v.2.2.7.1 (q-value < 0.01) with an input reference file. Peaks that were commonly found in both replicates of RBPJ ChIP-Seq and which did not overlap with ENCODE's backlisted regions (https://github.com/Boyle-Lab/Blacklist/) were used as the final peak set. Normalized coverage tracks were calculated using deepTools' bamCoverage function[19]. Snapshots were generated using GVIZ[20]. Motif identification was performed using the MEME suite[21].

CUT&Tag

CUT&Tag was performed with the CUT&Tag-IT™ Assay Kit from Active Motif (# 53160) accordingly to manufacturer's instructions and using an anti-H3K27ac antibody from Cell Signaling Technology (#8173). Libraries were quantified and sequencing was performed on a NovaSeq device at Novogene UK. Alignment and filtering of CUT&Tag data was performed identical to ChIP-Seq. Deeptools' bamCoverage function was used to compute normalized coverage tracks[19].

Usage of publicly available datasets

Gene expression data form CLL patients published by Lütge et al.[26] are available at European GenomephenomeArchive (EGA) under accession number EGAS00001001746. The original analysis is available at https://github.com/almutlue/transcriptome_cll. Statistical significance of differential expression between non-del(11q) and del(11q) patients was calculated using Wilcox test. To map the most accurate overlap possible with our data we did not apply a cut-off threshold to the significantly differentially expressed genes.

Gene expression data form the MILE-study was obtained from R2: Genomics Analysis and Visualization Platform (http://r2.amc.nl; R2 internal identifier: ps_avgpres_gse39671geo130_u133p2) using the DataGrabber tool of the website and not further modified.

***Ex vivo* viability experiments**

Viable cryopreserved primary CLL cells were thawed, resuspended in RPMI 1640 medium supplemented with 10% FBS and 1% penicillin/streptomycin (Gibco) and seeded at a concentration of 1 x 10^5 cells/well in a 96-well plate. Subsequently, cells were treated with DMSO (vehicle), AZ1 alone or in combination with nirogacestat, venetoclax or ibrutinib. After 24 hours, cell viability was measured in the form of ATP quantification by the Cell-Titer Glo Luminescent assay (Promega). All conditions were analyzed in triplicates and measurements from untreated cells were used for data normalization.

Statistical analyses

Statistical analysis was done using GraphPad PRISM (versions 9 and 10). Methods for significance testing were chosen depending on experimental design, number and variance of compared groups and data distribution as indicated in the respective figure legend. If applicable statistical tests were always performed two-tailed. Statistical tests were considered significant when the test resulted in a p-value <0.05.

Usage of AI assistants

To enhance the quality of our manuscript, A.S.E. and D.M. used support of the artificial intelligence tools perplexity.ai, ChatGPT (version 4o) from OpenAI and Claude3 Opus from Anthropic for literature review, brainstorming and text optimization following the guidelines of Elsevier, Springer, and the German Research Foundation (DFG).

Supplemental Figures

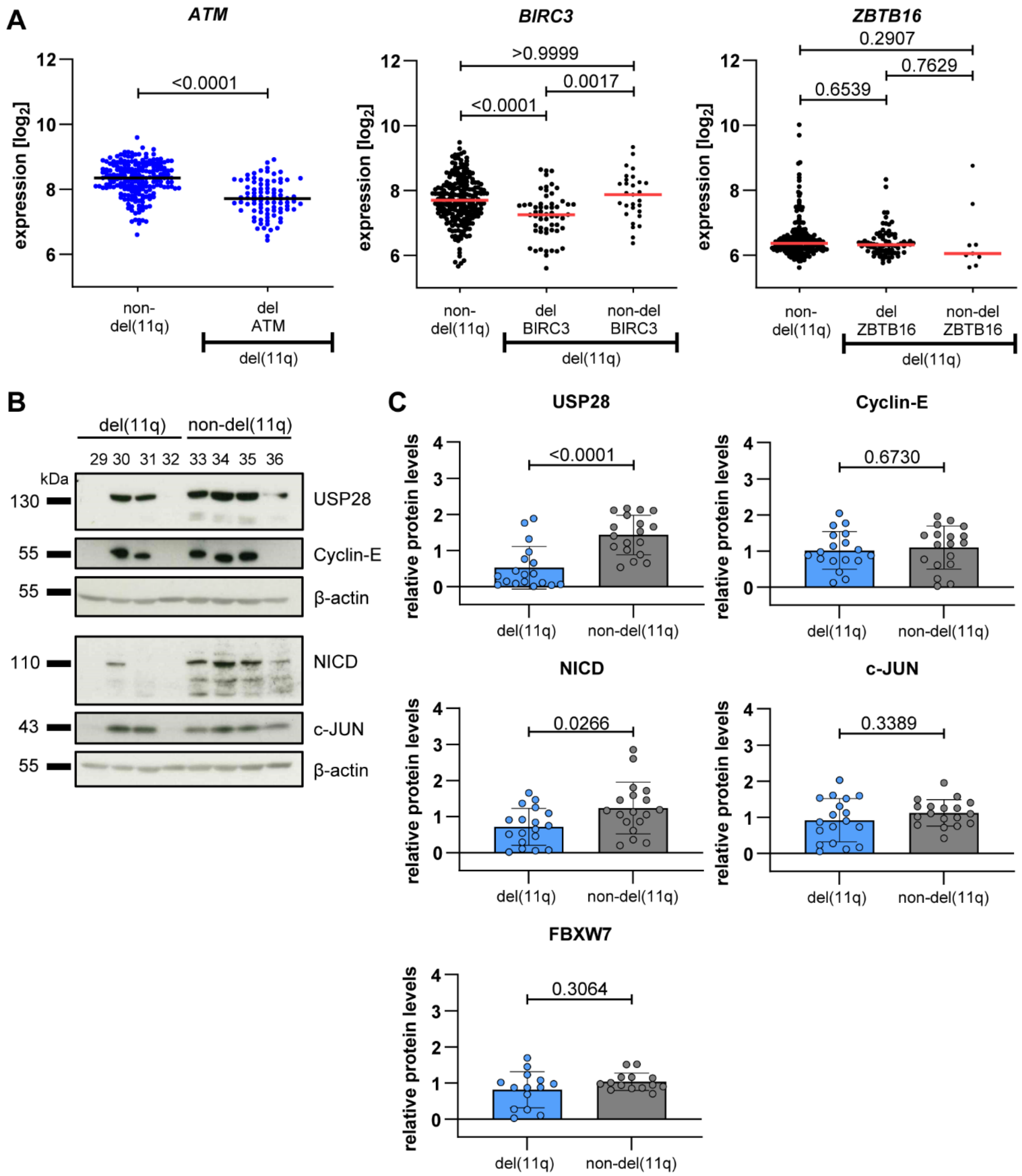

Supplemental Figure 1: del(11q) downregulates candidate genes and USP28 protein levels. (A) Expression in a clinical trial cohort of CLL patients (CLL8)[22,23] of *ATM*, *BIRC3* and *ZBTB16* in comparison between patients with (n=86) and without (n=199) del(11q). Statistical significance was determined by Mann-Whitney test for *ATM* and by the Kruskal-Wallis test followed by Dunn's multiple comparison test for *BIRC3* and *ZBTB16*. (B) Continuation of Figure 2A; Protein expression of USP28 and the FBXW7 target proteins Cyclin-E, NICD and c-JUN

in primary cells of CLL patients with and without del(11q). (C) Quantification of USP28, Cyclin-E, c-JUN, NICD protein levels from Figure 2 and (B) and FBXW7 protein levels from Figure 2 relative to β-actin. Statistical significance was assessed via unpaired Student's t test. Although not significant all proteins show the same trend of downregulation in del(11q) CLL cells.

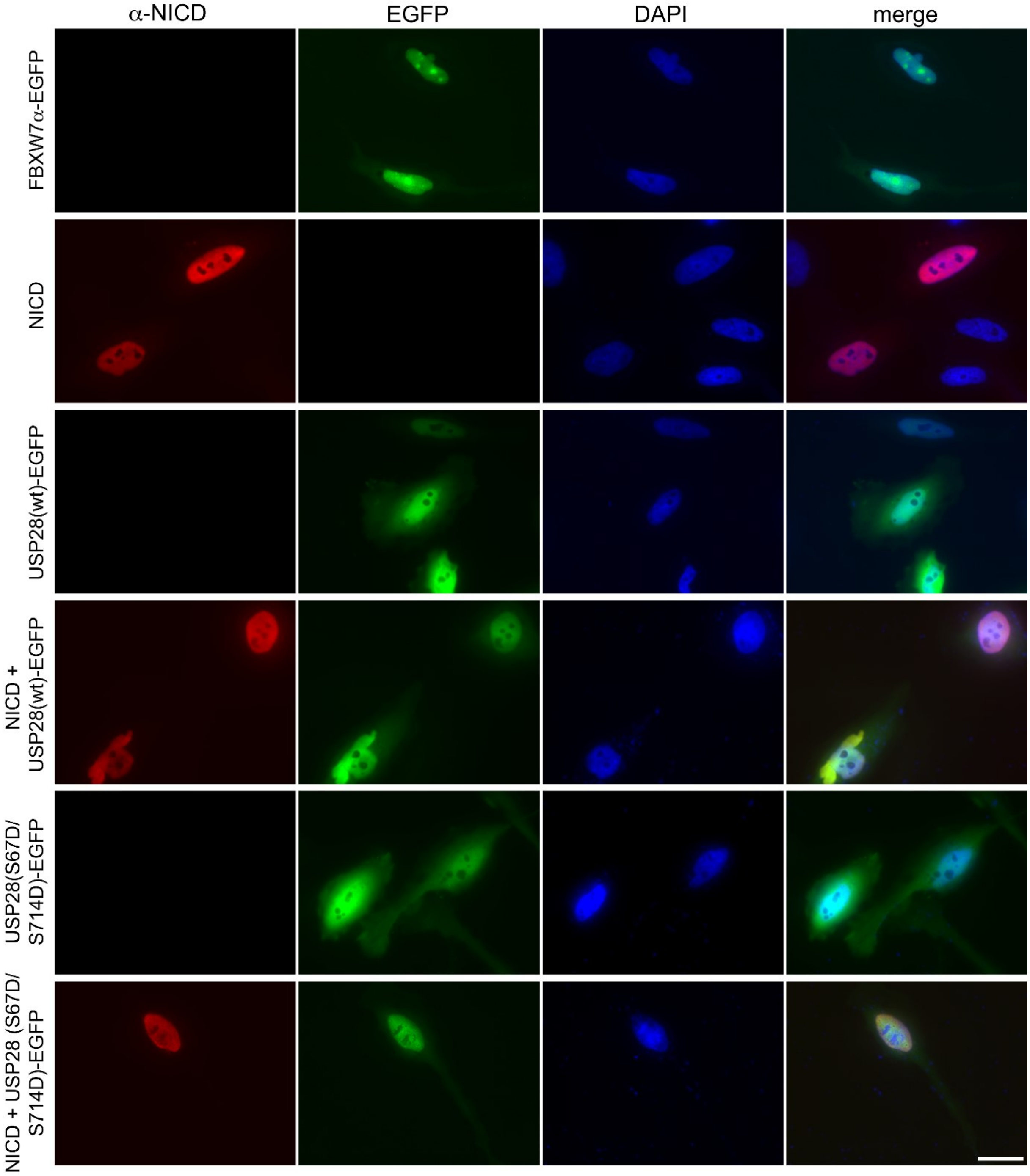

Supplemental Figure 2: FBXW7α, NICD, USP28(wt) and the phosphomimetic USP28 (S67D/S714D) are localized in the nucleus. Expression constructs listed on the left (250 ng)

were transfected into HeLa cells. After 24 h, NICD was visualized using specific antibodies (see materials and methods). FBXW7α and USP28 variants were detected by their EGFP fluorescence. DAPI staining was performed to show nuclei. Scale bar represents 10 µm.

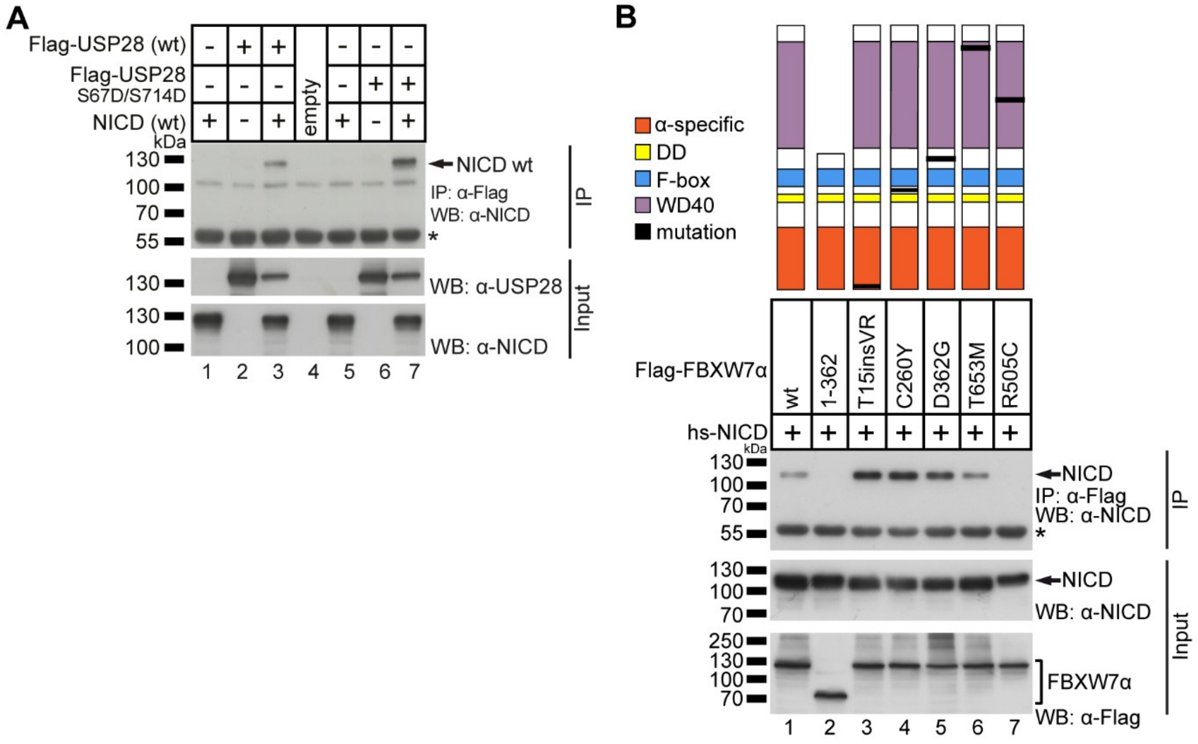

Supplemental Figure 3: USP28 (wt) and the phosphomimetic USP28 S67D/S714D, but not FBXW7 with truncated WD40 domain interact with NICD. (A) HEK293 cells were co-transfected with the indicated Flag-USP28(wt) or Flag-USP28 S67D/S714D and NICD (wt). Co-immunoprecipitation was performed 24 hours after transfection and analyzed via western blotting shown in the upper panel (IP). Expression of the proteins derived from the transfected constructs was detected via western blot shown in the two lower panels (Input). (B) HEK293 cells were transfected with the indicated Flag-tagged FBXW7α constructs with truncated or mutated WD40 domain which is important for interaction with NICD. Additionally, untagged NICD wt was transfected. Co-immunoprecipitation was performed 24 hours after transfection and analyzed via western blot shown in the upper panel (IP). The expression of the protein derived from the transfected constructs is shown in the lower panels (Input). * marks the heavy

fragment of the anti-Flag antibody used for IP. Western blots are representative for at least three independently performed experiments. WB, western blot; IP, immunoprecipitation.

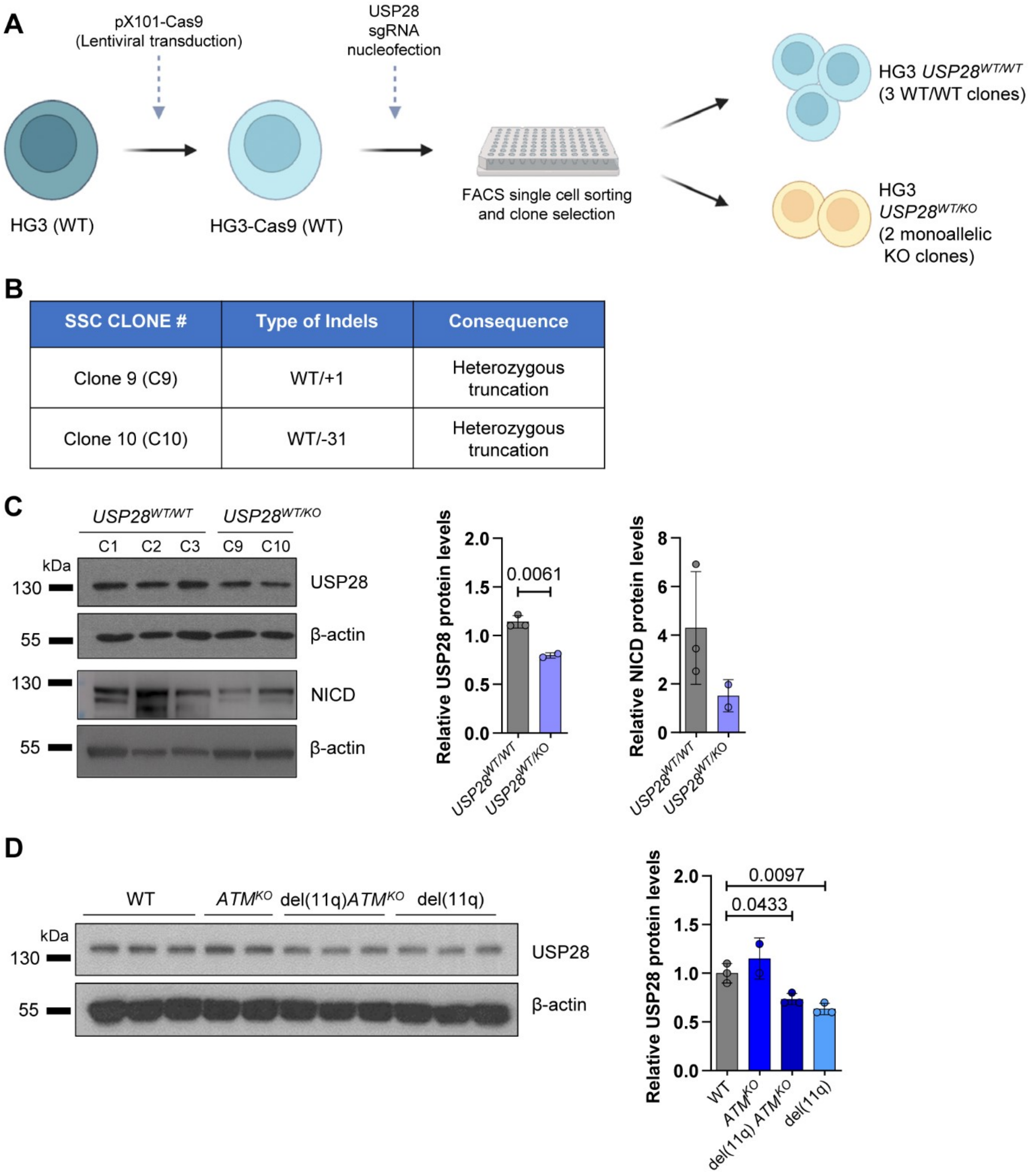

Supplemental Figure 4: CRISPR/Cas9-mediated deletion of 11q and heterozygous *USP28* knockout reduce USP28 protein levels. (A) Workflow of *USP28* knockout cell line generation. The CLL cell line HG3 is lentivirally transduced to express the Cas9 enzyme. After nucleofection with *USP28* sgRNAs and clone selection via single cell sorting three HG3

$USP28^{WT/WT}$ and two HG3 $USP28^{WT/KO}$ clones were obtained. (B) Sanger sequencing characterized heterozygous truncating mutations (indels) in the *USP28* target sequence. (C) Left panel: Western Blot of the different HG3 clones confirms lower USP28 and NICD expression in $USP28^{WT/KO}$ clones. Right panel: Quantification of USP28 and NICD protein levels in the HG3 $USP28^{WT/WT}$ and $USP28^{WT/KO}$ clones relative to β-actin. Statistical significance was assessed via unpaired Student's t test. (D) Left panel: USP28 protein levels in HG3 WT and the CRISPR/Cas9-generated ATM^{KO}, del(11q) ATM^{KO} and del(11q) HG3 clones. β-actin was used as loading control. Right panel: Quantification of USP28 protein levels in the HG3 WT and the CRISPR/Cas9-generated ATM^{KO}, del(11q) ATM^{KO} and del(11q) HG3 clones relative to β-actin. Statistical significance was assessed via one-way ANOVA. WT, wild type; KO, knockout; C, clone.

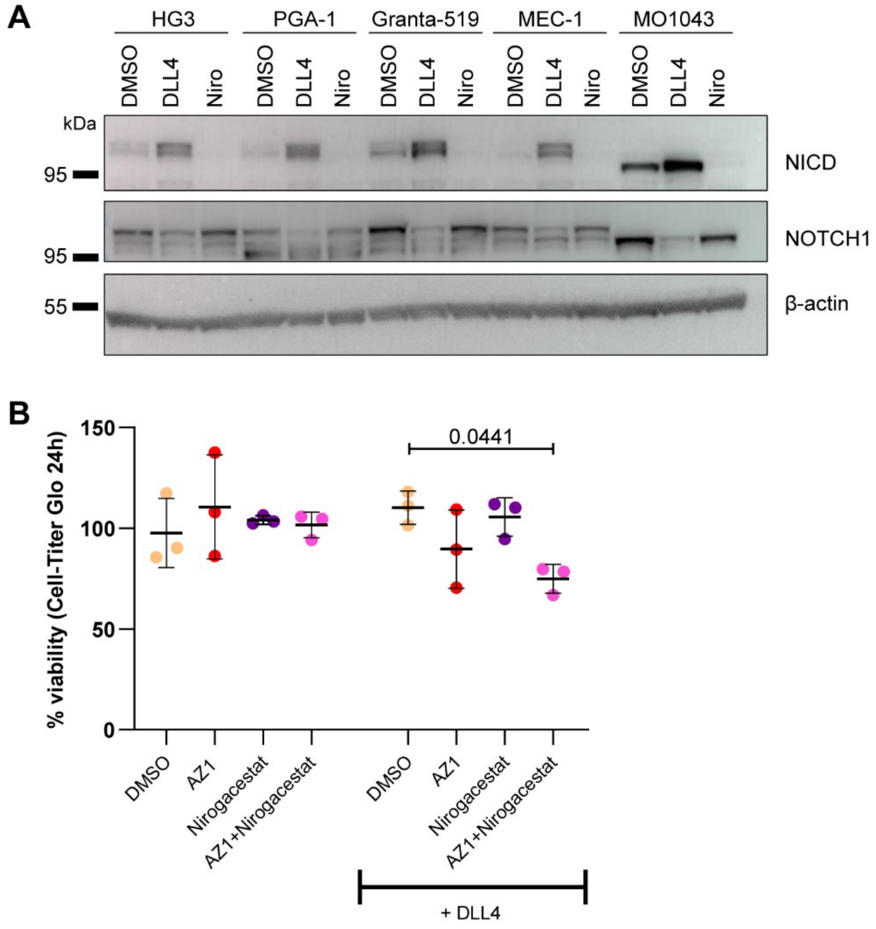

Supplemental Figure 5: NOTCH1 signaling is modulable in the CLL cell line HG3. (A) NICD and whole NOTCH1 protein levels in the cell lines HG3, PGA-1, Granta-519, MEC-1 and

MO1043 after 24 hours of treatment with DMSO as control, the NOTCH1 ligand DLL4 to stimulate NOTCH1 signaling or 1 μM of the γ-secretase inhibitor nirogacestat (Niro) to inhibit NOTCH1 signaling. β-actin was used as loading control. (B) Cell viability analysis by ATP quantification (Cell-Titer Glo) of HG3 cells with or without DLL4 stimulation 24 hours after treatment with DMSO, 10 μM AZ1, 1 μM nirogacestat or the combination of AZ1 and nirogacestat. Statistical significance was assessed via one-way ANOVA followed by Holm-Šídák's multiple comparisons test.

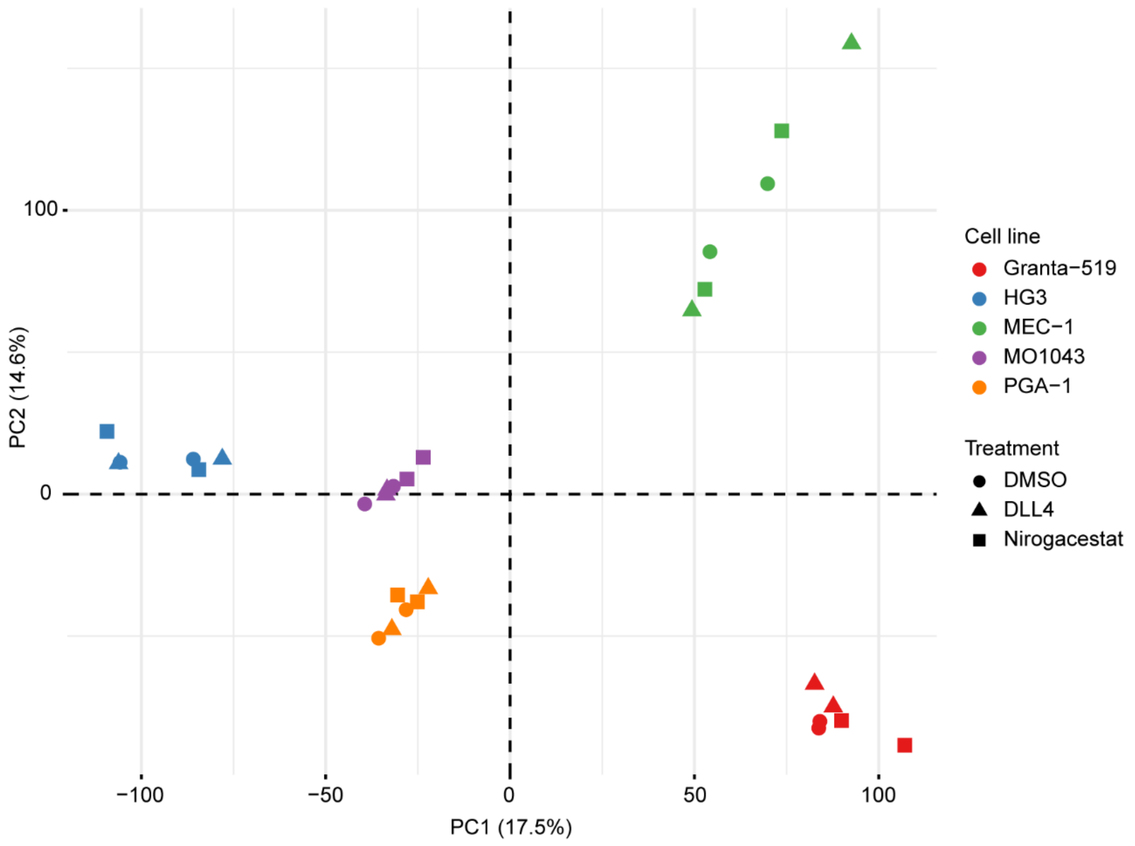

Supplemental Figure 6: Principal component analysis shows transcriptional variability among CLL and MCL cell lines. Principal Component Analysis of RNA-Seq data (FPKM values) from the cell lines HG3, PGA-1, Granta-519, MEC-1 and MO1043 treated for 24 hours with DMSO, DLL4 or nirogacestat. The plot shows PC1 and PC2, which explain 17.5% and 14.6% of the variance, respectively. Samples are color-coded by cell line, and shapes represent treatments. Each treatment was performed in duplicate.

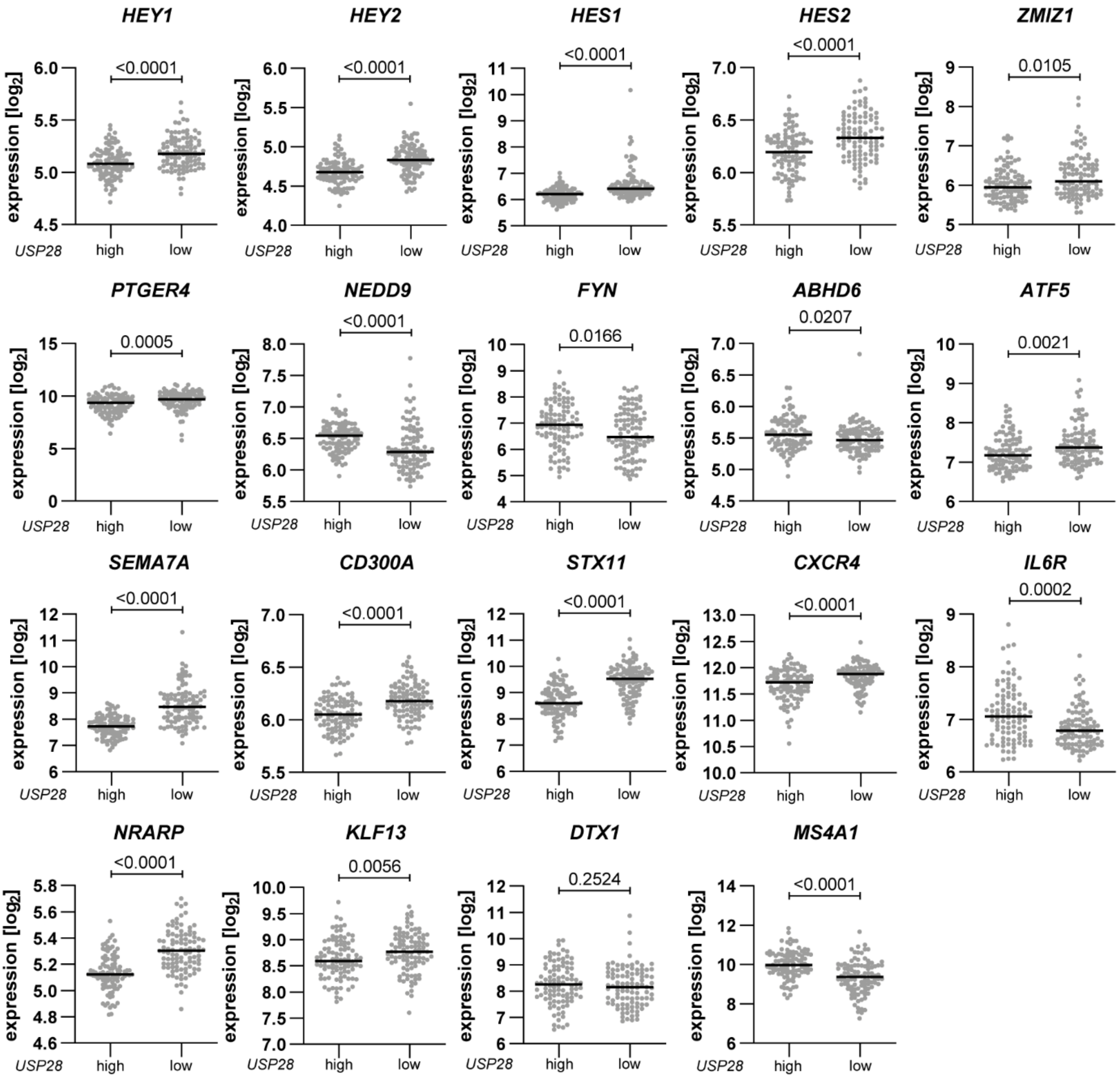

Supplemental Figure 7: NOTCH1 target gene expression is affected by *USP28* levels in non-del(11q) CLL patients. Gene expression levels of NOTCH1 target genes in CLL patients without del(11q) stratified into *USP28* high (n=99) and *USP28* low (n=100) at the median of *USP28* expression. The analyzed genes were selected based on a verification of CLL specific NOTCH1 target genes by Close et al.[6] upon the data sets by Fabbri et al.[22] and Ryan et al.[23]. Lines depict mean. Statistical significance was assessed via Mann-Whitney test.

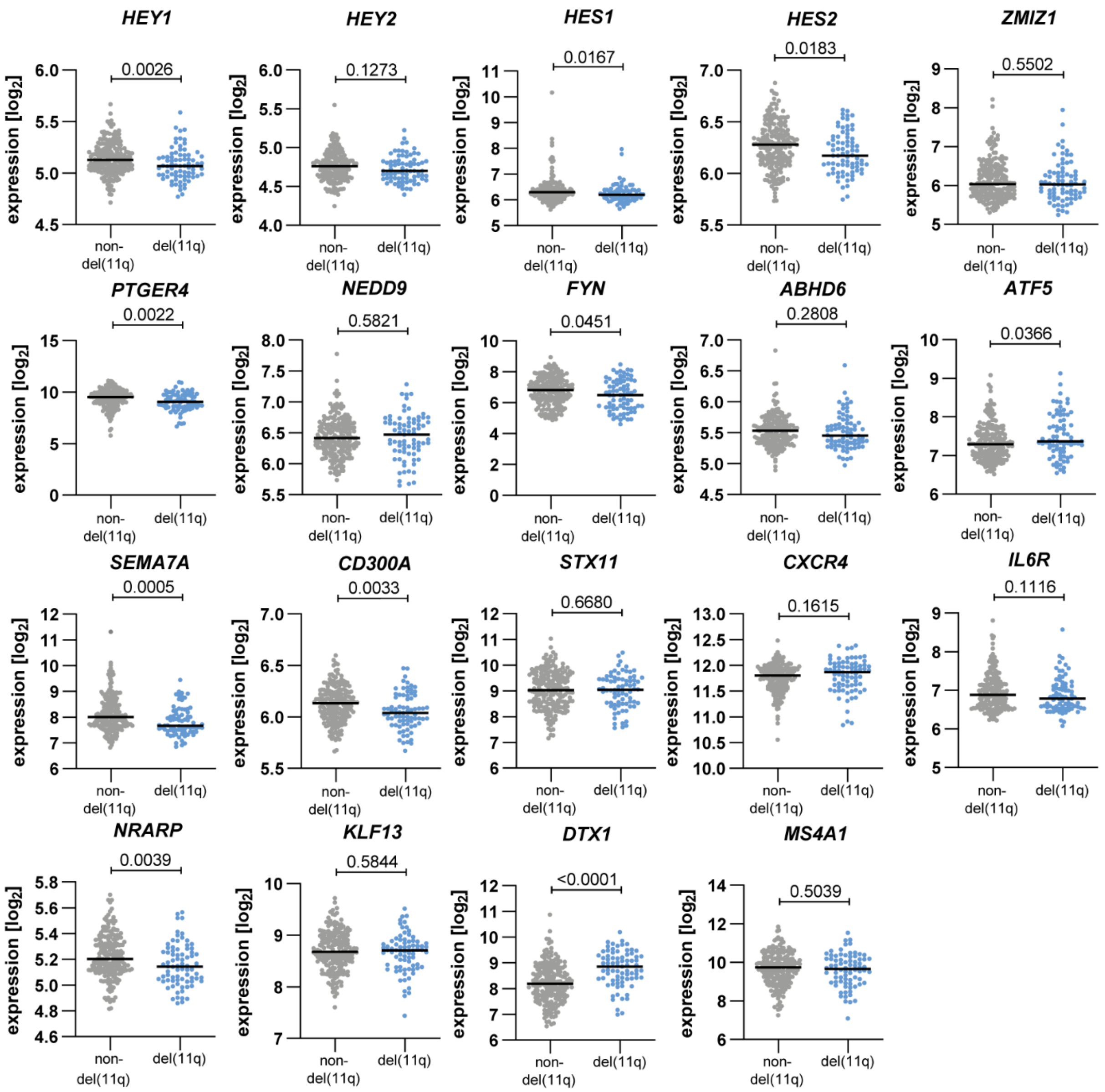

Supplemental Figure 8: NOTCH1 target gene expression is affected by del(11q). Gene expression levels of NOTCH1 target genes in CLL patients without del(11q) (non-del(11q), n=199) and del(11q) (n=86) patients with *USP28* deletion via del(11q) from the CLL8 cohort[24,25]. The analyzed genes were selected based on a verification of CLL specific NOTCH1 target genes by Close et al.[6] upon the data sets by Fabbri et al.[22] and Ryan et al.[23] Lines depict mean. Statistical significance was assessed via Mann-Whitney test.

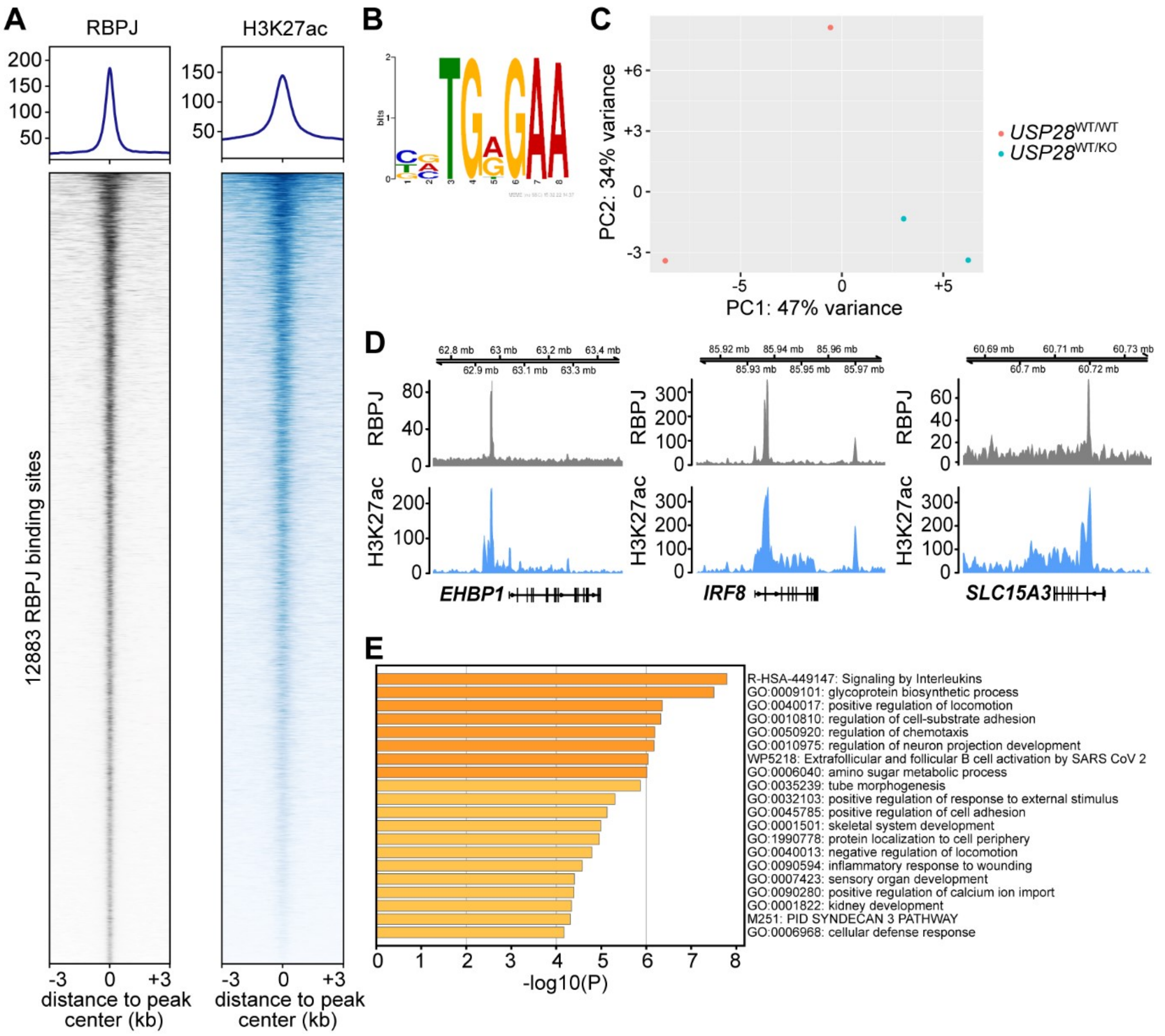

Supplemental Figure 9: RBPJ Chromatin-Immunoprecipitation sequencing identifies *bona fide* NOTCH1 target genes in HG3 cells. (A) HG3 wt CLL cells were analyzed by ChIP-Seq using an antibody versus RBPJ and CUT&Tag using an antibody versus H3K27ac. Heatmap shows the 12 883 RBPJ binding sites that were identified (left) and the enrichment of H3K27ac sites at the RBPJ binding sites (right). (B) RBPJ binding motif that is enriched among the RBPJ binding sites identified in HG3 cells. (C) Principal component plot showing similarities in gene expression between RNA-Seq in *USP28*$^{WT/WT}$ (n=2, red) and *USP28*$^{WT/KO}$ (n=2, green) HG3 clones. (D) Representative snapshots showing peaks of RBPJ binding (upper panel, grey) and H3K27 acetylation (lower panel, blue) at the gene loci of *EHBP1*, *IRF8* and *SLC15A3.* (E) Top 20 pathways enriched among the 129 genes significantly dysregulated

between *USP28*$^{WT/WT}$ and *USP28*$^{WT/KO}$ cells and bound by RBPJ (Figure 5A). Pathways were identified by Metascape[17].

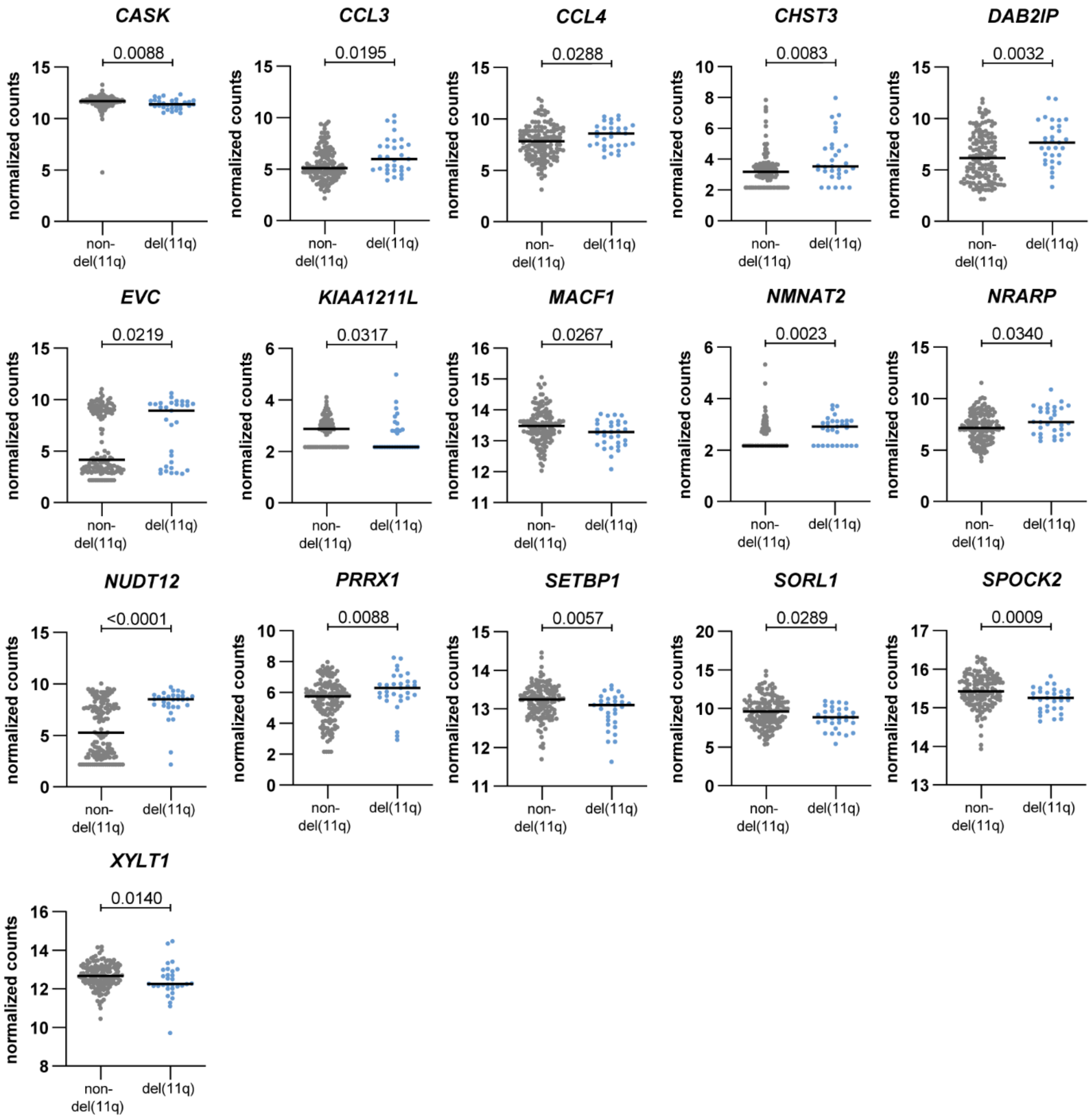

Supplemental Figure 10: RBPJ-bound and USP28 dysregulated genes are affected by del(11q) in CLL patients. Gene expression levels of NOTCH1 target genes that were identified via RBPJ-ChIP-Seq and differentially expressed in *USP28*$^{WT/KO}$ HG3 cells in CLL patients without del(11q) (non-del(11q), n=138) and del(11q) patients (n=31) from the patient cohort analyzed by Lütge et al.[26] (n=27). Lines depict mean. Statistical significance was

assessed via Mann-Whitney test reporting exact p-values. The remaining overlapping genes are shown in Figure 6 and Supplemental Figure 11.

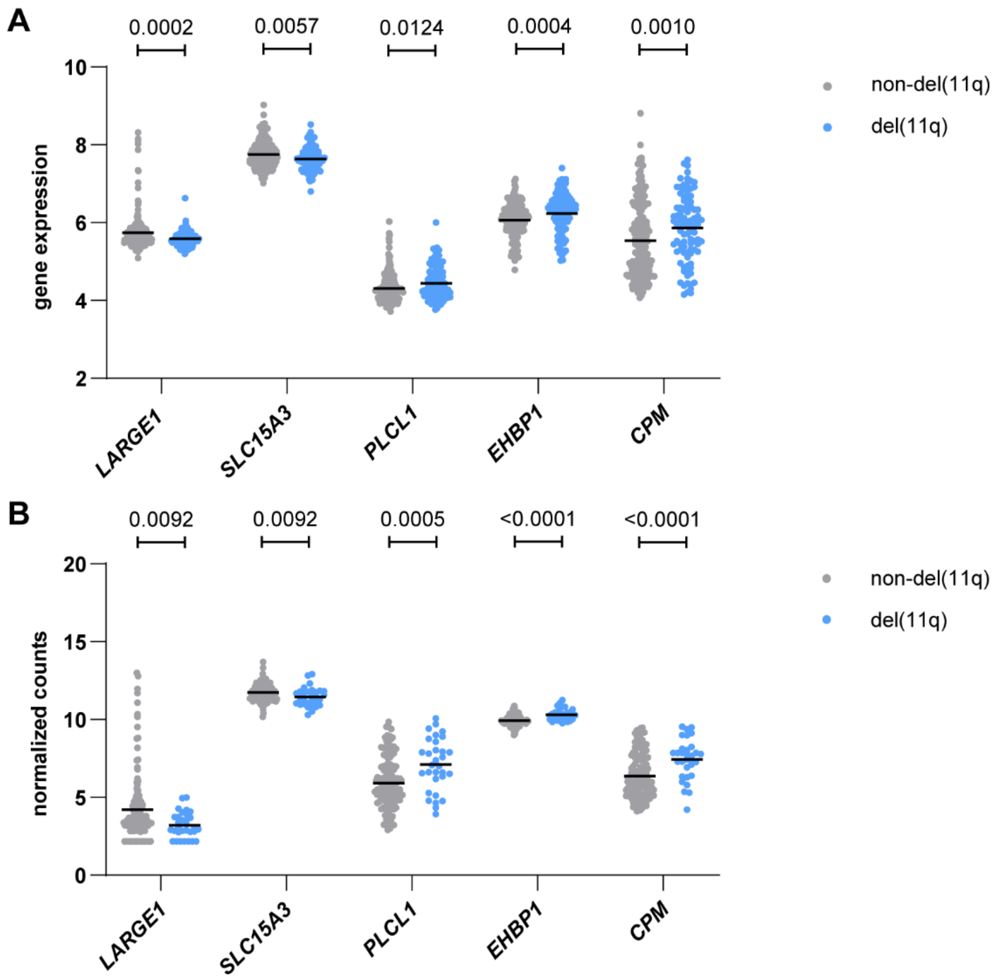

Supplemental Figure 11: *LARGE1*, *SLC15A3*, *PLCL1*, *EHBP1* and *CPM* are commonly dysregulated by del(11q) CLL. NOTCH1 target gene expression in the data sets of (A) non-del(11q) (n=199) and del(11q) (n=86) CLL patients from the CLL8 gene expression study[25], and (B) non-del(11q) (n=138) and del(11q) (n=31) patients analyzed in Lütge et al.[26] Statistical significance was assessed via Mann-Whitney test. Lines depict mean.

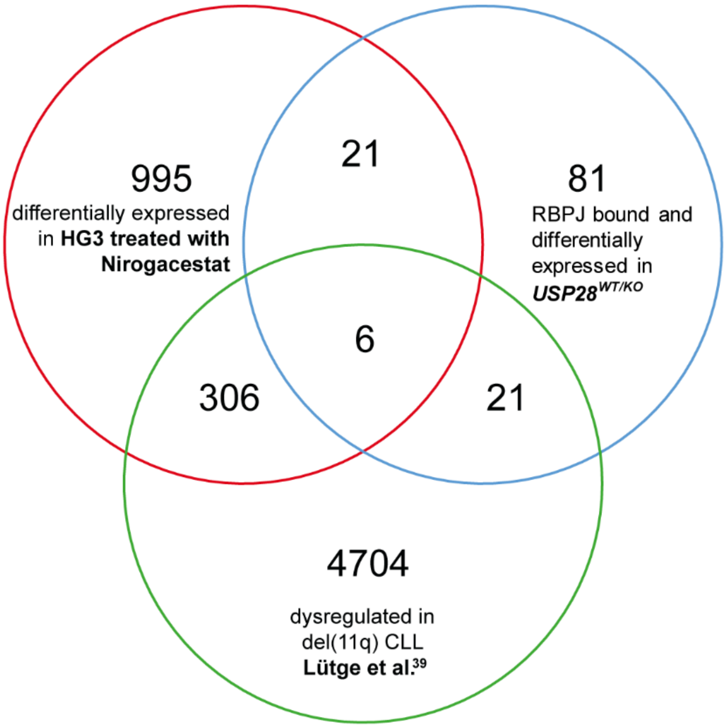

Supplemental Figure 12: A set of 6 NOTCH1 target genes is commonly dysregulated by 11q deletion, heterozygous loss of USP28 and NOTCH1 inhibition in HG3 cells. Venn diagram illustrating the intersection of: RBPJ-bound, differentially expressed genes in *USP28*$^{WT/KO}$ (n=129; blue circle), significantly differentially expressed genes in del(11q) CLL patients from Lütge et al.[26] (n=5037; green circle) and significantly differentially expressed genes in HG3 cells treated with the γ-secretase inhibitor nirogacestat to inhibit NOTCH1 signaling (n=1328; red circle). The overlap between the three datasets defines a set of 6 NOTCH1 target genes which are not only dysregulated by heterozygous deletion of USP28 but also directly by inhibition of NOTCH1 signaling.

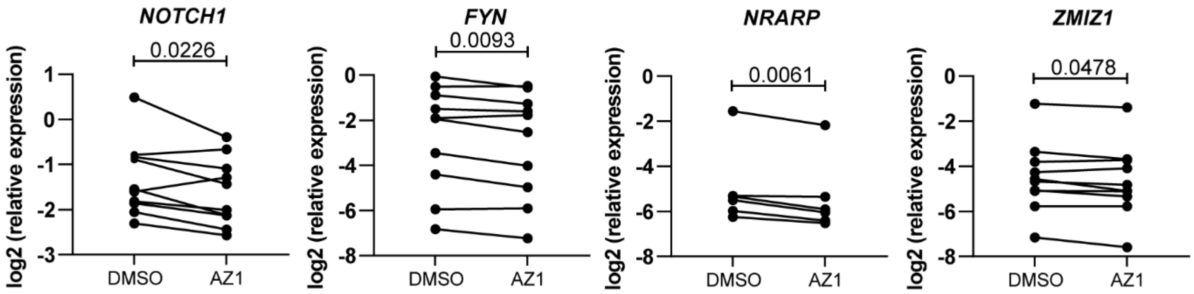

Supplemental Figure 13: NOTCH1 target genes are downregulated upon USP28 inhibition via AZ1 treatment. Relative expression of *NOTCH1* and its target genes *FYN, ZMIZ1* and *NRARP* in primary CLL samples (n=10) upon treatment with DMSO or AZ1 (10 µM; 24 hours). These genes have been previously validated to be CLL specific NOTCH1 target genes by Close et al.[6] upon the data sets by Fabbri et al.[22] and Ryan et al.[23]. Expression is relative to three housekeeping genes and was log2 transformed. Dots represent single patient samples; lines connect gene expression of DMSO and AZ1 treated samples from the same patient. Patient characteristics of the analyzed samples are available in Supplemental Table 3. *NRARP*, expression was detectable in both samples of only 6/10 patients. Statistical significance was assessed via paired Student's t test.

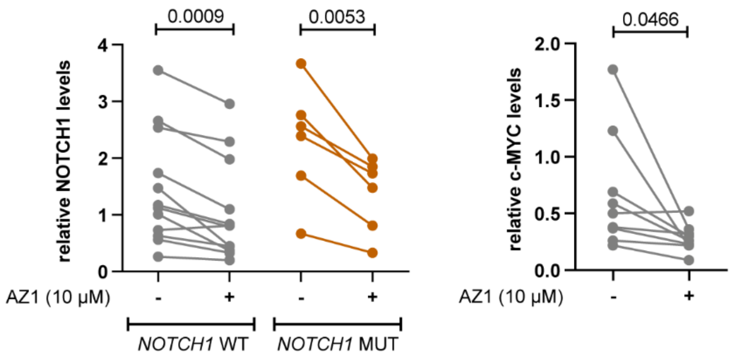

Supplemental Figure 14: NOTCH1 and c-MYC protein levels are decreased in primary CLL cells treated with AZ1. Relative NOTCH1 and c-MYC protein levels in DMSO-treated vs AZ1-treated primary CLL cells without (grey) and with (brown) *NOTCH1* mutations. The quantification is based on the Western blot in Figure 7B. Statistical significance was assessed via paired Student's t test.

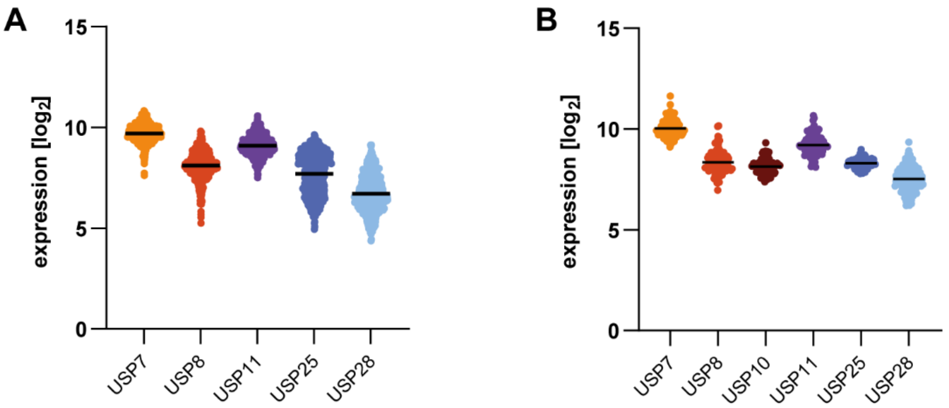

C

mean log2(norm. counts	USP7	USP8	USP10	USP11	USP25	USP28
HG3 *USP28* *WT/WT* (n=2)	12.682 SD 0.032	11.052 SD 0.136	12.095 SD 0.044	10.553 SD 0.139	10.499 SD 0.206	10.346 SD 0.020
HG3 *USP28* *WT/KO* (n=2)	12.676 SD 0.048	10.952 SD 0.005	12.267 SD 0.024	10.709 SD 0.100	10.655 SD 0.094	10.127 SD 0.026
adj. p-value	0.8937	0.4904	0.0644	0.3382	0.4694	**0.0133**

Supplemental Figure 15: Gene expression of NICD-associated deubiquitinases and the USP28 homolog USP25 in CLL patients and HG3-derived cell lines. Gene expression of *USP7, USP8, USP10, USP11, USP25* and *USP28* in CLL patients of the (A) CLL8 gene expression study[25] (n = 726) and the (B) MILE-study (n = 130; obtained from R2: Genomics Analysis and Visualization Platform (http://r2.amc.nl); R2 internal identifier: ps_avgpres_gse39671geo130_u133p2). *USP10* expression data was not available from the CLL8 study. (C) Mean expression values and their standard deviation (SD) of *USP7, USP8, USP10, USP11, USP25* and *USP28* in the HG3 *USP28*$^{WT/KO}$ (clones 9 and 10) and *USP28*$^{WT/WT}$ cell lines (clones 1 and 2). Comparing gene expression of the deubiquitinases between the cell lines shows a significant difference for *USP28* (p = 0.0133). Statistical significance was assessed via two-way ANOVA followed by Šídák's multiple comparisons test.

Supplemental Table 1. IGHV status, cytogenetics assessed via FISH and *TP53* status of CLL patients used for western blots in Figure 2 and Supplemental Figure 1B.

Sample ID	Sex	IGHV status	Cytogenetics (FISH)	*TP53* status	Sample used in
1	M	N/A	del(13q), del(11q)	Unmutated	Fig.2
2	M	Unmutated	del(13q), del(11q)	Unmutated	Fig.2
3	M	N/A	del(13q), del(11q), del(14q)	Mutated	Fig.2
4	M	Unmutated	del(13q), del(11q)	Unmutated	Fig.2
5	M	Unmutated	del(13q),del(11q)	Unmutated	Fig.2
6	M	Unmutated	del(13q), del(11q)	Unmutated	Fig.2
7	F	Unmutated	del(13q), del(11q)	Unmutated	Fig.2
8	M	Mutated	del(13q)	Unmutated	Fig.2
9	F	Mutated	normal karyotype	Unmutated	Fig.2
10	F	Unmutated	del(13q)	Unmutated	Fig.2
11	M	Mutated	del(13q)	Unmutated	Fig.2
12	M	Mutated	del(13q)	Unmutated	Fig.2
13	M	Unmutated	normal karyotype	Unmutated	Fig.2
14	F	Mutated	del(13q)	Unmutated	Fig.2
15	M	N/A	del(11q)	Unmutated	Fig.2
16	M	Unmutated	bidel(13q), del(11q)	Unmutated	Fig.2
17	M	Unmutated	del(11q)	Unmutated	Fig.2
18	F	Unmutated	del(13q), del(11q)	Unmutated	Fig.2
19	F	Unmutated	del(11q)	Unmutated	Fig.2
20	F	Unmutated	del(13q), del(11q)	Unmutated	Fig.2
21	M	Unmutated	del(13q), del(11q)	Unmutated	Fig.2
22	M	Mutated	del(13q)	Unmutated	Fig.2

23	M	Unmutated	del(13q)	Unmutated	Fig.2
24	M	Mutated	del(13q)	Unmutated	Fig.2
25	F	Mutated	del(13q)	Unmutated	Fig.2
26	F	Mutated	del(13q)	Unmutated	Fig.2
27	M	Mutated	del(17p)	Mutated	Fig.2
28	M	Mutated	del(13q)	Unmutated	Fig.2
29	M	Mutated	del(11q)	Unmutated	Suppl. Fig.1
30	F	Unmutated	del(11q)	Unmutated	Suppl. Fig.1
31	M	Unmutated	del(11q)	Unmutated	Suppl. Fig.1
32	M	Mutated	del(13q), del(11q)	Unmutated	Suppl. Fig.1
33	F	Mutated	del(13q)	Unmutated	Suppl. Fig.1
34	F	Unmutated	del(13q)	Unmutated	Suppl. Fig.1
35	M	Unmutated	del(13q)	Unmutated	Suppl. Fig.1
36	M	Mutated	normal karyotype	Unmutated	Suppl. Fig.1

N/A, not assessed; FISH, fluorescence in-situ hybridization

Supplemental Table 2. Enrichment of the KEGG Notch signaling pathway gene set (ko04330) in the transcriptome of HG3 cells treated with DLL4 or Nirogacestat (Niro).

compared samples	ID	description	set size	enrichment score	NES	p value	q value	enriched genes
**HG3 vs HG3_DLL4**	ko04330 (KEGG)	Notch signaling pathway	50	-0.5469	-1.8207E+14	0.0021	0.0862	*HEY1; CREBBP; NCSTN; NBPF26; TLE3; DVL1; NOTCH1; DLL1; NewGene_9312; PSEN1; TLE1; HEY2; DTX4; PSEN2; DLL4; NOTCH2; CTBP2*
**HG3 vs HG3_Niro**	ko04330 (KEGG)	Notch signaling pathway	50	-0.5078	-1.8523E+14	0.0025	0.0467	*PSEN1; CREBBP; TLE1; NCSTN; NOTCH2NLA; EP300; NewGene_9312; DLL4; HEY2; DTX4; PSEN2; NOTCH2; CTBP2; AD000671.1*

NES, normalized enrichment score

Supplemental Table 3. IGHV status, cytogenetics assessed via FISH and *TP53* status of CLL patients used for analysis of NOTCH1 target genes upon AZ1 treatment Supplemental Figure 13.

Sample	Sex	IGHV status	Cytogenetics (FISH)	*TP53* status
CLL1	F	Mutated	del(13q)	Unmutated
CLL2	M	Unmutated	normal karyotype	Unmutated
CLL3	M	Mutated	del(13q)	Unmutated
CLL4	M	Unmutated	del(17q)	Mutated
CLL5	F	Mutated	normal karyotype	Unmutated
CLL6	M	Mutated	del(13q)	Unmutated
CLL7	F	Mutated	normal karyotype	Unmutated
CLL8	M	Mutated	del(13q)	Unmutated
CLL9	M	Unmutated	normal karyotype	Unmutated
CLL10	F	Mutated	del(13q)	Unmutated

FISH, fluorescence in-situ hybridization

Supplemental Table 4. Patient characteristics of primary CLL cells treated with AZ1 and analyzed via western blotting in Figure 7B and cell viability analysis in Figure 8C.

Sample ID	Sex	IGHV status	Cytogenetics (FISH)	NOTCH1 pathway mutations	Type of mutation	Other mutated genes
#1	M	Unmutated	del(17p)			*TP53, DMD*
#2	F	Mutated	del(13q)			*KLHL6, KRAS*
#3	F	Mutated	del(13q), del(11q)			0
#4	F	Unmutated	+(12), t(14;18)	*NOTCH1*	p.P2514fs	0
#5	M	Unmutated	+(12)	*FBXW7* (4)	p.E569X; p.X708G; p.K444E; p.R465H	0
#6	F	Unmutated	del(13q)	*NOTCH1*	p.P2514fs	*XPO1, NRAS*
#7	M	Unmutated	del(11q)			*XPO1, FUBP1*
#8	M	Mutated	del(13q)			0
#9	F	Unmutated	normal karyotype			*ATM, BRAF, MAP2K1, PTPN11, EGR2*

#10	M	Unmutated	del(13q), del(11q)			*ATM, NFKBIE*
#11	M	Unmutated	normal karyotype	*NOTCH1* (2)	p.P2514fs; p.Q2406X	*SF3B1*
#12	F	Unmutated	del(13q)	*MED12, NOTCH1*	p.G44C; p.P2514fs	*SF3B1, RPS15*
#13	M	Mutated	del(13q), +(12)			*IGLL5, DDX3X*
#14	M	N/A	+(12)			*HIST1H1E, NFKBIE, KRAS, IGLL5*
#15	F	Mutated	normal karyotype			*SF3B1*
#16	F	Mutated	del(13q)			0
#17	M	Mutated	del(13q)			*TP53, ARID1B, CCND2, CHD2*
#18	M	Unmutated	del(13q)	*NOTCH1*	p.P2514fs	*XPO1, MGA*

N/A, not assessed; FISH, fluorescence in-situ hybridization

Supplemental Table 5. IGHV status, cytogenetics assessed via FISH and *TP53* status of CLL patients used for cell viability analysis in Figure 8B.

Sample	Sex	IGHV status	Cytogenetics (FISH)	*NOTCH1* status	*TP53* status
wt1	M	Mutated	del(13q)	Unmutated	Unmutated
wt2	M	Mutated	del(13q)	Unmutated	Unmutated
wt3	N/A	Unmutated	del(11q)	Unmutated	Unmutated
wt4	F	Unmutated	del(11q), del(13q)	Unmutated	Unmutated
wt5	M	Unmutated	del(13q)	Unmutated	Unmutated
wt6	M	Unmutated	del(14q)	Unmutated	Unmutated
wt7	F	Mutated	del(13q), del(14q)	Unmutated	Unmutated
wt8	M	Mutated	del(13q)	Unmutated	Unmutated
wt9	F	Mutated	del(13q)	Unmutated	Unmutated
wt10	M	N/A	del(17p)	Unmutated	Mutated
wt11	F	Mutated	normal karyotype	Unmutated	Unmutated
wt12	M	Mutated	del(13q)	Unmutated	Unmutated
wt13	F	Mutated	del(13q)	Unmutated	Unmutated
wt14	F	Unmutated	del(11q), del(13q), del(14q)	Unmutated	Unmutated
wt15	M	Unmutated	del(11q), del(13q)	Unmutated	Unmutated
wt16	M	Unmutated	del(13q)	Unmutated	Unmutated
wt17	F	Mutated	++(13q)	Unmutated	Unmutated
mut1	M	Unmutated	del(13q), del(14q)	Mutated	Unmutated
mut2	N/A	Unmutated	+(12)	Mutated	Unmutated
mut3	N/A	Unmutated	normal karyotype	Mutated	Unmutated
mut4	N/A	Unmutated	del(13q)	Mutated	Unmutated
mut5	N/A	Unmutated	del(13q), del(14q)	Mutated	Unmutated

mut6	N/A	Mutated	del(13q)	Mutated	Unmutated
mut7	N/A	Unmutated	del(13q)	Mutated	Unmutated
mut8	N/A	Unmutated	del(11q), del(13q)	Mutated	Unmutated
mut9	N/A	Unmutated	normal karyotype	Mutated	Unmutated
mut10	N/A	Unmutated	normal karyotype	Mutated	Unmutated
mut11	N/A	Unmutated	+(12)	Mutated	Unmutated
mut12	N/A	Unmutated	+(12)	Mutated	Unmutated
mut13	N/A	Unmutated	normal karyotype	Mutated	Unmutated

N/A, not assessed; FISH, fluorescence in-situ hybridization

References

1. Kawata A, Han T, Dadey B, Weier HU, Okazaki M, Yokota S, et al. Establishment and characterization of the tumors of chronic lymphocytic leukemia cell line in nude and SCID mice. Leuk Res. 1993;17(10):883-94.
2. Richter K, Paakkola T, Mennerich D, Kubaichuk K, Konzack A, Ali-Kippari H, et al. USP28 deficiency promotes breast and liver carcinogenesis as well as tumor angiogenesis in a HIF-independent manner. Mol Cancer Res. 2018;16(6):1000-1012.
3. Durkin ME, Qian X, Popescu NC, Lowy DR. Isolation of mouse embryo fibroblasts. Bio Protoc. 2013;3(18):e908.
4. López-Guerra M, Xargay-Torrent S, Fuentes P, Roldán J, González-Farré B, Rosich L, et al. Specific NOTCH1 antibody targets DLL4-induced proliferation, migration, and angiogenesis in NOTCH1-mutated CLL cells. Oncogene. 2020 Feb;39(6):1185-1197.
5. Zhang D, Zaugg K, Mak TW, Elledge SJ. A role for the deubiquitinating enzyme USP28 in control of the DNA-damage response. Cell. 2006;126(3):529-42.
6. Close V, Close W, Kugler SJ, Reichenzeller M, Yosifov DY, Bloehdorn J, et al. FBXW7 mutations reduce binding of NOTCH1, leading to cleaved NOTCH1 accumulation and target gene activation in CLL. Blood. 2019;133(8):830-839.
7. Flügel D, Görlach A, Kietzmann T. GSK-3β regulates cell growth, migration, and angiogenesis via Fbw7 and USP28-dependent degradation of HIF-1α. Blood. 2012;119(5):1292-301.
8. Oswald F, Täuber B, Dobner T, Bourteele S, Kostezka U, Adler G, et al. p300 acts as a transcriptional coactivator for mammalian Notch-1. Mol Cell Biol. 2001;21(22):7761-74.
9. Schneider CA, Rasband WS, Eliceiri KW. NIH Image to ImageJ: 25 years of image analysis. Nature Methods. 2012;9:671-675.
10. Ruijter JM, Ramakers C, Hoogaars WM, Karlen Y, Bakker O, van den Hoff MJ, et al. Amplification efficiency: linking baseline and bias in the analysis of quantitative PCR data. Nucleic Acids Res. 2009;37(6):e45.

11. Dobin A, Davis CA, Schlesinger F, Drenkow J, Zaleski C, Jha S, et al. STAR: ultrafast universal RNA-seq aligner. Bioinformatics. 2013;29(1):15-21.
12. Putri GH, Anders S, Pyl PT, Pimanda JE, Zanini F. Analysing high-throughput sequencing data in Python with HTSeq 2.0. Bioinformatics. 2022;38(10):2943-2945
13. Love MI, Huber W, Anders S. Moderated estimation of fold change and dispersion for RNA-seq data with DESeq2. Genome Biol. 2014;15(12):550.
14. Wickham H. ggplot2: Elegant graphics for data analysis. New York: Springer; 2016.
15. Kim D, Langmead B, Salzberg SL. HISAT: a fast spliced aligner with low memory requirements. Nat Methods. 2015;12(4):357-60.
16. Lawrence M, Huber W, Pagès H, Aboyoun P, Carlson M, Gentleman R, et al. Software for computing and annotating genomic ranges. PLoS Comput Biol. 2013;9(8):e1003118.
17. Zhou Y, Zhou B, Pache L, Chang M, Khodabakhshi AH, Tanaseichuk O, et al. Metascape provides a biologist-oriented resource for the analysis of systems-level datasets. Nat Commun. 2019;10(1):1523.
18. Ferrante F, Giaimo BD, Bartkuhn M, Zimmermann T, Close V, Mertens D, et al. HDAC3 functions as a positive regulator in Notch signal transduction. Nucleic Acids Res. 2020;48(7):3496-3512.
19. Ramírez F, Dündar F, Diehl S, Grüning BA, Manke T. deepTools: a flexible platform for exploring deep-sequencing data. Nucleic Acids Res. 2014;42(Web Server issue):W187-91.
20. Hahne F, Ivanek R. Visualizing Genomic Data Using Gviz and Bioconductor. Methods Mol Biol. 2016;1418:335-51.
21. Bailey TL, Johnson J, Grant CE, Noble WS. The MEME Suite. Nucleic Acids Res. 2015;43(W1):W39-49.
22. Fabbri G, Holmes AB, Viganotti M, Scuoppo C, Belver L, Herranz D, et al. Common nonmutational NOTCH1 activation in chronic lymphocytic leukemia. Proc Natl Acad Sci U S A. 2017;114(14):E2911-E2919.

23. Ryan RJH, Petrovic J, Rausch DM, Zhou Y, Lareai CA, Kluk MJ, et al. A B cell regulome links Notch to downstream oncogenic pathways in small B cell lymphomas. Cell Rep. 2017;21(3):784-797.
24. Edelmann J, Holzmann K, Miller F, Winkler D, Bühler A, Zenz T, et al. High-resolution genomic profiling of chronic lymphocytic leukemia reveals new recurrent genomic alterations. Blood. 2012;120(24):4783-94.
25. Bloehdorn J, Braun A, Taylor-Weiner AN, Jebaraj BMC, Robrecht S, Krzykalla J, et al. Multi-platform profiling characterizes molecular subgroups and resistance networks in chronic lymphocytic leukemia. Nat Commun. 2021;12(1):5395.
26. Lütge A, Lu J, Hüllein J, Walther T, Sellner L, Wu B, et al. Subgroup-specific gene expression profiles and mixed epistasis in chronic lymphocytic leukemia. Haematologica. 2023;108(10):2664-2676.
